# Modeling the effect of age in T1-2 breast cancer using the SEER database

**DOI:** 10.1186/1471-2407-5-130

**Published:** 2005-10-08

**Authors:** Patricia Tai, Gábor Cserni, Jan Van De Steene, Georges Vlastos, Mia Voordeckers, Melanie Royce, Sang-Joon Lee, Vincent Vinh-Hung, Guy Storme

**Affiliations:** 1 University of Saskatchewan, Faculty of Medicine, Department of Radiation Oncology, Regina, Canada; 2 Bács-Kiskun County Teaching Hospital, Surgical Pathology, Kecskemét, Hungary; 3 AZ-VUB, Oncologisch Centrum, and BISI-VUB, Computer Science and Medical Informatics, Jette, Belgium; 4 Geneva University Hospitals, Department of Gynecology and Obstetrics, Senology and Gynecologic Oncology Unit, Geneva, Switzerland; 5 University of New Mexico, Cancer Research and Treatment Center, Albuquerque, New Mexico, USA; 6 University of New Mexico, Department of Internal Medicine, Division of Epidemiology and Biostatistics, Albuquerque, New Mexico, USA

## Abstract

**Background:**

Modeling the relationship between age and mortality for breast cancer patients may have important prognostic and therapeutic implications.

**Methods:**

Data from 9 registries of the Surveillance, Epidemiology, and End Results Program (SEER) of the United States were used. This study employed proportional hazards to model mortality in women with T1-2 breast cancers. The residuals of the model were used to examine the effect of age on mortality. This procedure was applied to node-negative (N0) and node-positive (N+) patients. All causes mortality and breast cancer specific mortality were evaluated.

**Results:**

The relationship between age and mortality is biphasic. For both N0 and N+ patients among the T1-2 group, the analysis suggested two age components. One component is linear and corresponds to a natural increase of mortality with each year of age. The other component is quasi-quadratic and is centered around age 50. This component contributes to an increased risk of mortality as age increases beyond 50. It suggests a hormonally related process: the farther from menopause in either direction, the more prognosis is adversely influenced by the quasi-quadratic component. There is a complex relationship between hormone receptor status and other prognostic factors, like age.

**Conclusion:**

The present analysis confirms the findings of many epidemiological and clinical trials that the relationship between age and mortality is biphasic. Compared with older patients, young women experience an abnormally high risk of death. Among elderly patients, the risk of death from breast cancer does not decrease with increasing age. These facts are important in the discussion of options for adjuvant treatment with breast cancer patients.

## Background

In many clinical situations, age is an important determinant of treatment decision in breast cancer. For example, after mastectomy, patients with T2 tumors and one to three positive nodes are at high risk of isolated loco-regional recurrences. Authors have advocated the routine use of postmastectomy radiotherapy in those patients who have T2 tumors and who are younger than 45 years [1]. In another study about close margins at mastectomy, the subgroup of patients aged 50 or younger with clinical T1-2 tumors and 0–3 positive nodes who have close (5 mm or less) or positive margins were at high risk (28% at 8 years) for chest wall recurrence regardless of adjuvant systemic therapy. Therefore, such patients should be considered for postmastectomy radiation [2]. Young women aged less than 45 should be regarded as high-risk patients, on the basis of age alone, and should be given adjuvant cytotoxic treatment [3]. The latter study showed a non-linear relationship between age and relative risk of dying.

At the other end of the age spectrum, breast cancers in elderly patients have been considered by some authors to exhibit a less aggressive behavior than in younger patients [4,5]. Other authors have argued that breast cancer does not become more indolent as age increases [6].

There are still controversial issues about the relationship between age and prognosis in breast cancer. Detailed analysis would be useful in order to provide more insight into this relationship. In the present study, we used proportional hazards to model the survival of T1-2, node-negative (N0) and node-positive (N+) breast cancer patients. Outcomes which we considered included all-cause mortality and cancer specific mortality from breast cancer. The primary aim of the study is to present how age relates with the risk of death. The secondary objective is to search for a simple algebraic representation of this relationship.

## Methods

The Surveillance, Epidemiology, and End Results Program (SEER) of the United States collected data about the incidence of cancer and related matters from 11 population-based registries [7]. The data extracted in this study was from 9 registries: San Francisco-Oakland, Connecticut, Metropolitan Detroit, Hawaii, Iowa, New Mexico, Seattle (Puget Sound), Utah, and Metropolitan Atlanta. Selected patients were women who were without previous history of cancer and presented with non-inflammatory invasive breast carcinoma, diagnosed and histologically confirmed pT1-2 pM0 between 1988 and 1997, and for whom curative surgery and axillary lymph node dissections were performed. In 1987, the American Joint Committee on Cancer (AJCC) staging defined pT1 tumors as 2 cm or less in greatest dimension, and pT2 tumor as more than 2 cm but not more than 5 cm in greatest dimension. These definitions did not change until 1997. Some records were rejected because of concerns about the quality of data: non-hospital based data records, uncertain sequence of treatment, unknown month of diagnosis and unknown race. Records with missing histological grade and receptor status were not excluded. Examination of statistical outliers excluded one case with 75 nodes involved. Events for the study were death from all causes and death from breast cancer. Follow-up cutoff date was December 31, 1999 as provided by the database.

In order to verify the linearity of the continuous variables, the martingale residuals (differences between observed and expected numbers of events) were used. The martingale residuals were examined by a non-parametric smoothing (fitting the scatter-plots of residuals) against the quantitative covariates of interest. The smoothing used a Poisson regression implementation of generalized additive model (GAM) [8]. The GAM procedure provided two outputs. One was the non-parametric smoothed curves approximating the residuals. The other was a significance test of the non-linearity of the curves. For the covariates that significantly departed from linearity, an iterative search was performed to identify parametric families of functions that approximated the curves. The criteria used to end the search were: [a] simple parametric expression, [b] the corresponding function introduced as a transform in the Cox model satisfying the GAM linearity test, and [c] without deteriorating the model fit as assessed by the sum of squares of "deviance residuals" [8]. If the transforms were valid, the graphical displays should be linear shapes, and the non-linearity test results should be non-significant. Finally, scaled Schoenfeld residuals were used to verify that the relative hazards were constant over time [9]. The hypothesis underlying this dual modeling approach was as follows. If the algebraic functions are valid, their use as plug-in transforms should appropriately linearize the functional forms of the covariates of interest. Other information about the implementation of these procedures have been described earlier [10-12].

The analysis was applied first to node-negative cases ("training set") in order to find a simple expression of the functional form which relates age to mortality. The functional form obtained from node-negative cases was then applied to node-positive cases ("validation set"). In addition to the validation with the same transformation which was obtained for node-negative patients, a further iterative search was performed in order to improve the fit for node-positive patients.

This analysis was applied also to a European dataset, the German Breast Cancer Study Group (GBSG-2), in which the outcome studied was disease-free survival [14]. From a data analysis perspective, this GBSG-2 dataset is a very different database of 686 patients containing some extreme observations. One case had 51 involved nodes (range for other patients 1–38), and another case had a tumor size of 120 mm (range for other patients 3–100). There were 299 events (either recurrence of disease or death) in this German database.

The statistical analyses were performed with Splus (Insightful Corporation, Seattle, WA, USA) statistical software. Parametric fitting of curves used TableCurve 2D (Systat Software Inc, Richmond, CA, USA).

## Results

There are 83,804 T1-2 cases (58,139 N0 and 25,665 N+, mean: 4 nodes involved, range: 1–48) available for analysis from the SEER database. Table 1 shows the characteristics of the patients. This table has been presented elsewhere [13]. Except for 28 additional cases (because of updated registration), there are no noticeable differences in the distribution of the characteristics. Table 2 shows the results of proportional hazards models in N0 and N+ groups, without using transforms for covariates. The supplemental Table 2b (Additional file 1) shows results of the check for Cox proportional hazards for all covariates. Note that some P-values are very small because of the very large size of the data. The rho-values (slope) indicate very small departures from the assumption of proportional hazards.

**Figure 1:**
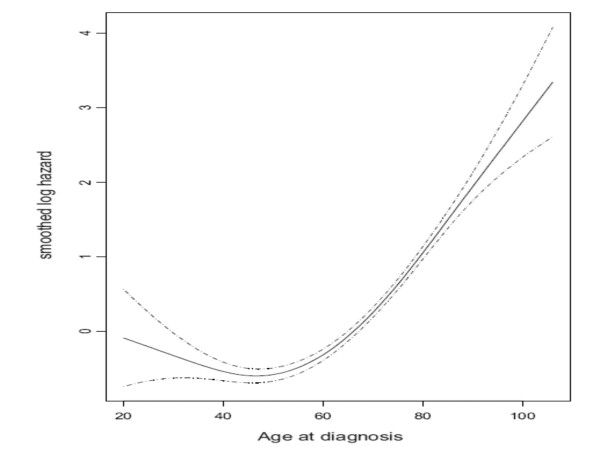
Mortality for all causes as a function of age for N0 patients. Dotted lines: twice-standard-error.

**Figure 2:**
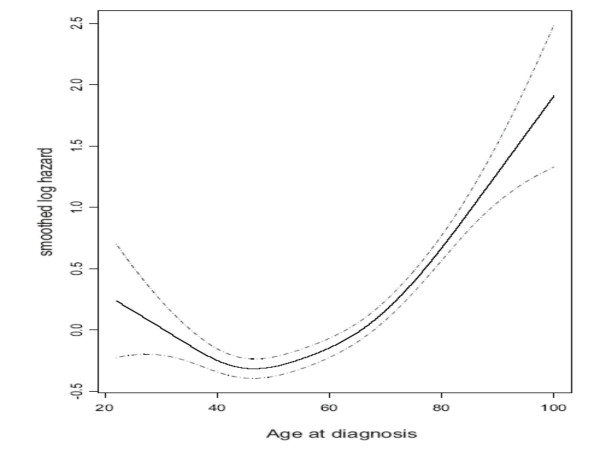
Mortality for all causes as a function of age for N+ patients. Dotted lines: twice-standard-error.

Figures 1 and 2 show graphically the effect of age on the log hazard ratio for death from *all causes*, for N0 and N+ patients, respectively. Both curves have similar U-shapes. The mortality is lowest for patients about 50 years of age at diagnosis. The mortality increases the farther away from 50 years of age at diagnosis, for both younger and older patients.

The shape of the smoothed curve for age suggests the use of a quadratic function. A fractional polynomial analogous to Sauerbrei and Royston [14], but with different exponents, combining a linear term **(age) **and a quasi-quadratic term **|age-50|**^1.5^, i.e. **age**+ **|age-50|**^1.5^, provides a good fit and passes the test of linearity (Chi-square = 6.530, P = 0.089) in N0 patients (Table 3).

We note that the age transform derived from node-negative cases does not provide a perfect linearization in N+ patients (Table 3). A better linearization in N+ patients was obtained by replacing the 1.5 exponent with 1.8, though without improving global model fit (Table 3).

The proportional hazard check for age shows a deviation from the assumption of constant hazard (Table 3). The "rho" values are positive when considering overall mortality, i.e. an increasing risk of death with longer follow-up. The values are negative when considering breast cancer specific mortality, i.e. a decreasing risk of breast cancer death with longer follow-up.

The age transforms suggest two components in the effect of age. One component is linear (linear for the log hazard ratio, i.e. exponential for the hazard ratio) and corresponds to a natural increase in mortality with each year of age. The other component is quasi-quadratic and is centered around age 50. It contributes to an increased risk of mortality as age increases beyond 50. It suggests a hormonally related process, not pre- versus post-menopausal, but *perimenopausal *versus *non-perimenopausal (premenopausal + postmenopausal)*. The further age at diagnosis is from the age at menopause, the more prognosis is influenced by the quasi-quadratic component.

The results display a complex functional form of the effect of age on mortality. The curves clearly highlight the biological anomaly that younger patients experience the same relative mortality risk from all causes as do older patients. Figures 1 and 2 show that a 30-year old patient has a risk of death almost equal to a 60-year old patient.

**Figure 3:**
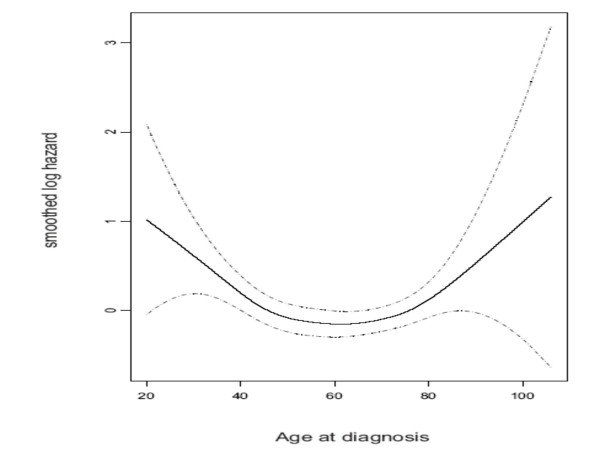
Breast cancer mortality as a function of age for N0 patients. Dotted lines: twice-standard-error.

**Figure 4:**
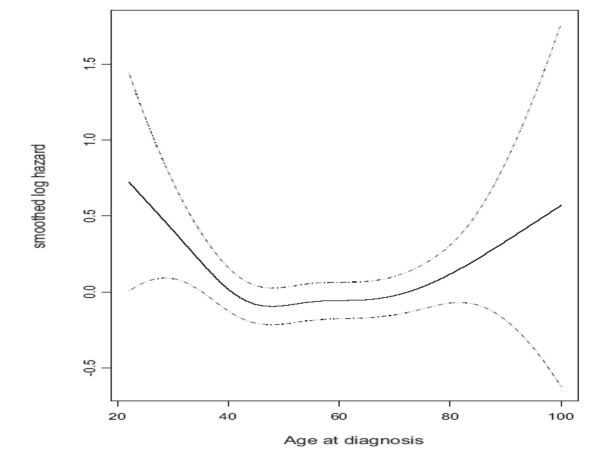
Breast cancer mortality as a function of age for N+ patients. Dotted lines: twice-standard-error.

The marked increase in mortality risk at older ages is attributable to the increased risk of death from causes other than breast cancer (co-morbidity). It should be noted that breast cancer does not become less virulent in older patients. An increase in the risk of death from breast cancer associated with older age was observed both in N0 and in N+ patients (Figures 3 and 4).

The German Breast Cancer Study Group GBSG-2 dataset [14] is a separate database of 686 patients. Using the GAM procedure on the GBSG-2 data, age was significantly non-linear (Chi^2 ^= 31.744, 3 degrees of freedom, P < 0.000001). The age transforms improved the linearity for the age variable, and also improved the proportional hazards model (Table 4).

## Discussion

In studies addressing the effect of age on breast cancer, several authors have reported a biphasic mortality [15-19]. This large study concurs with others in the literature. As in any modeling, the validity and the utility of the model may be questioned. Data from the GBSG-2 study were considered for verification of the model. The GBSG-2 study differs from the present SEER study in several respects. This German study was a prospective controlled clinical trial about the adjuvant treatment of node-positive breast cancer patients. Inclusion of patients was not restricted by tumor size. Histopathological classification and grading were performed centrally by one reference pathologist. The GBSG-2 data have been extensively investigated for the effect of age on the prognosis of breast cancer [20]. The GBSG-2 data thus provide an indication of the capability of our results to be extrapolated to a different population. It is also complementary, since the SEER has no data on recurrence and can provide no information on disease-free survival.

Applying different methods to estimate the effect of age on event-free survival of breast cancer (linear, categorization based on cutpoints, classification and regression trees, quadratic, fractional polynomial, cubic splines), Hollaender found that all methods showed a decrease in risk with increasing age up to 45–50 years [20]. A slight increase in risk was observed for older patients in the GBSG-2 data. Taking into account the wide confidence intervals for ages older than 80 years, our Figure 4 for node-positive breast cancer specific survival shows a good concordance with the node-positive GBSG-2 event-free survival.

Regarding the proportional hazards assumption, Hollaender noted that assuming a linear risk function, a small correlation value rho of 0.147 was obtained [20]. Our result for the GBSG-2 data shows the value of rho to be 0.131 (Table 4). The small difference is attributable to the incorporation of different covariates to our proportional hazards model (additional file 2 "outputgbsg2.doc"). For the SEER data, the rho values are smaller (Table 3).

Our results are also in keeping with a closely related investigation of the SEER data in which a group of 4,616 patients 35 years old or younger was compared to a group of 20,319 patients aged 50–55 years [21]. The authors observed that younger breast cancer patients had poorer survival explained in part by presentation with later stage disease and more aggressive tumors, in terms of grade and receptor status. But the known factors could not account for the remaining unexplained difference in survival. In contradiction, recently Rapiti et al have argued that age is not an independent prognostic factor when accounting for breast tumor characteristics and treatment [22]. However, this latter study included only 82 patients who were 35 years old or younger.

**Figure 5:**
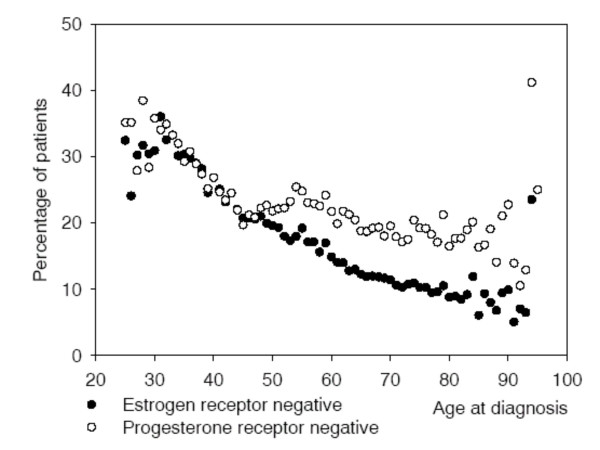
Distribution of hormone receptor status by age.

In order to try to understand the biphasic mortality, we looked at hormonal status and treatments of the patients. The age of 50 corresponds to the menopause. A large proportion of younger women were estrogen receptor (ER) negative (Figure 5). The proportion of ER-negative patients decreases with increasing age without any inflection. On the other hand, the proportion of progesterone receptor (PR) negative patients increases at age 50 then slowly decreases again. The reporting of hormonal receptor status is incomplete in SEER (~33–35% missing data).

**Figure 6:**
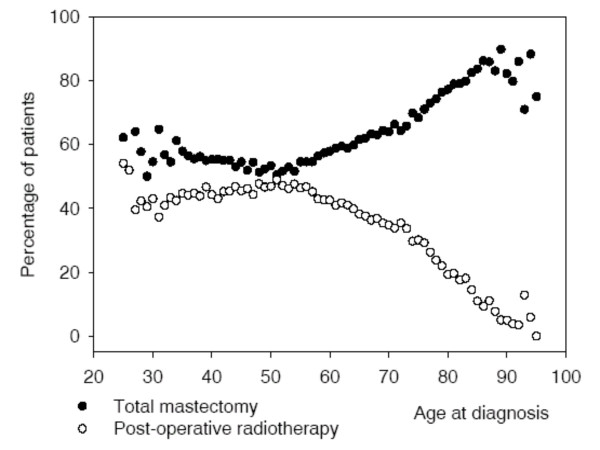
Distribution of treatment by age.

Data on systemic treatment were not available from the SEER database, but the types of surgery and radiotherapy were provided. Mastectomy was performed less frequently on younger patients, but increased markedly among older patients. Post-operative radiotherapy was given less frequently at both ends of the age spectrum; somewhat less frequently in the young and considerably less frequently in the elderly patients (Figure 6). Researchers have reported under-treatment of elderly patients and this fact may account in part for the poor prognosis in the elderly [23-25]. Whether hormonal status or type of treatment or other factors may explain the biphasic mortality will need to be researched.

There are several limitations in the present analysis. The data are retrospective. Several orders of statistically significant interactions have not been incorporated in the models. Receiving systemic treatment is a particularly important prognostic factor in younger patients [3], but data on systemic treatment were not available for analysis.

Despite the limitations and regardless of the modeling, our major finding is that the relationship of age and mortality is biphasic. Such a finding has been described by many other authors [16,17,20,26]. It is important to remember this biphasic relationship when analyzing the effect of age on patients with breast cancer. Otherwise, there is a substantial risk of misinterpreting results when age is inappropriately categorized [26] or inappropriately modeled. (Table 2 would suggest erroneously almost no effect of age on mortality). Taking into account the full shape of the relationship between age and breast cancer specific mortality, we conclude that: 1) young women experience a much higher risk of death than do older patients; 2) among elderly patients, the risk of death from breast cancer does not decrease with increasing age. These are two facts that should be remembered by those when discussing adjuvant treatment with breast cancer patients.

## Conclusion

The present analysis confirms that the relationship between age and mortality is biphasic. It is important that clinical research takes this relationship into account.

## Competing interests

The author(s) declare that they have no competing interests.

## Authors' contributions

PT, VVH: writing the manuscript; SJL, VVH: data analysis;

GCs, JVDS, GV, MR, MV, GS: concept, design, and drafting the manuscript.

All authors read and approved the final manuscript.

## Pre-publication history

The pre-publication history for this paper can be accessed here:



## Supplementary Material

Additional File 1 **Check of the Cox proportional hazards assumption.** Click here for file

Additional File 3 **Output of Cox, GAM and cox zph.** Click here for file

Additional File 2 **GBSG-2 data set.** Click here for file

## Figures and Tables

**Table 1:** Characteristics of the patients.

**Characteristics of patients**	**Total No.**	**% of all cases**	**Total N(0)**	**% of all cases**	**Total N(+)**	**% of all cases**
Patient number	83804	100.0%	58139	69.4%	25665	30.6%

SEER area:						
East (Connecticut, Detroit, Atlanta)	31795	37.9%	21683	25.9%	10112	12.1%
Central (Iowa, New Mexico, Utah)	20459	24.4%	14202	16.9%	6257	7.5%
West (San Francisco-Oakland, Hawaii, Seattle)	31550	37.6%	22254	26.6%	9296	11.1%
						
Year of diagnosis:						
1988–89	15390	18.4%	10424	12.4%	4966	5.9%
1990–91	16660	19.9%	11589	13.8%	5071	6.1%
1992–93	17042	20.3%	11894	14.2%	5148	6.1%
1994–95	17072	20.4%	11897	14.2%	5175	6.2%
1996–97	17640	21.0%	12335	14.7%	5305	6.3%
						
Age at diagnosis (years):						
20–39	5869	7.0%	3396	4.1%	2473	3.0%
40–49	16231	19.4%	10262	12.2%	5969	7.1%
50–59	18051	21.5%	12362	14.8%	5689	6.8%
60–69	20703	24.7%	15110	18.0%	5593	6.7%
70–79	17161	20.5%	12802	15.3%	4359	5.2%
80+	5789	6.9%	4207	5.0%	1582	1.9%
						
Race:						
White and Other	78025	93.1%	54572	65.1%	23453	28.0%
Black	5779	6.9%	3567	4.3%	2212	2.6%
						
Marital status at diagnosis:						
Single, widowed, other	33224	39.6%	23171	27.6%	10053	12.0%
Married	50580	60.4%	34968	41.7%	15612	18.6%
						
Topography:						
Inner quadrant	12537	15.0%	9726	11.6%	2811	3.4%
Others	71267	85.0%	48413	57.8%	22854	27.3%
						
Histology:						
Ductal	64370	76.8%	43924	52.4%	20446	24.4%
Others	19434	23.2%	14215	17.0%	5219	6.2%
						
Grade:						
Poor/undifferentiated	25161	30.0%	15265	18.2%	9896	11.8%
Others	58643	70.0%	42874	51.2%	15769	18.8%
						
ER/PR status (+ includes unspecified):						
ER+ and PR+	63306	75.5%	44332	52.9%	18974	22.6%
ER+ and PR-	7001	8.4%	4852	5.8%	2149	2.6%
ER- and PR+	2554	3.0%	1696	2.0%	858	1.0%
ER- and PR-	10943	13.1%	7259	8.7%	3684	4.4%
						
Tumor size (mm) (T stage):						
< = 5 (T1a)	3349	4.0%	3069	3.7%	280	0.3%
> 5 and < = 10 (T1b)	16997	20.3%	14691	17.5%	2306	2.8%
> 10 and < = 20 (T1c)	36754	43.9%	26443	31.6%	10311	12.3%
> 20 and < = 50 (T2)	26704	31.9%	13936	16.6%	12768	15.2%
						
Number of nodes examined:						
1–9	14225	17.0%	10525	12.6%	3700	4.4%
10–14	27004	32.2%	19210	22.9%	7794	9.3%
15–19	23102	27.6%	15820	18.9%	7282	8.7%
20+	19473	23.2%	12584	15.0%	6889	8.2%
						
Number of nodes involved:						
0	58139	69.4%	58139	69.4%	0	0.0%
1–3	16778	20.0%	0	0.0%	16778	20.0%
4+	8887	10.6%	0	0.0%	8887	10.6%
						
Breast conserving surgery/Radiotherapy						
no/no	46862	55.9%	31752	37.9%	15110	18.0%
no/yes	3972	4.7%	1024	1.2%	2948	3.5%
yes/no	4438	5.3%	2946	3.5%	1492	1.8%
yes/yes	28532	34.0%	22417	26.7%	6115	7.3%

ER = estrogen receptor, N(0) = node negative, N(+) = node positive, PR = progesterone receptor, SEER = Surveillance, Epidemiology, and End Results Program.

**Table 2:** Results of Cox proportional hazards models without transform. Hazard ratios (95% confidence intervals), values > 1 indicate increased risk of death.

	**Overall mortality**		**Breast cancer specific mortality**	
	
	**N0**	**N+**	**N0**	**N+**
SEER central area	1.00 (0.95–1.05)	0.93 (0.87–0.98)	1.03 (0.94–1.13)	0.92 (0.86–0.99)
SEER western area	0.92 (0.88–0.97)	0.87 (0.82–0.92)	0.82 (0.75–0.90)	0.86 (0.80–0.91)
Race black	1.38 (1.27–1.49)	1.42 (1.31–1.52)	1.37 (1.21–1.55)	1.44 (1.31–1.57)
Married	0.75 (0.72–0.78)	0.82 (0.78–0.86)	0.88 (0.81–0.95)	0.91 (0.86–0.97)
Inner quadrant	1.09 (1.04–1.15)	1.16 (1.08–1.24)	1.32 (1.21–1.44)	1.3 (1.19–1.41)
Ductal histology	1.14 (1.09–1.2)	1.11 (1.04–1.17)	1.44 (1.31–1.58)	1.12 (1.05–1.21)
ER negative	1.39 (1.28–1.5)	1.52 (1.41–1.64)	1.58 (1.4–1.78)	1.58 (1.44–1.73)
PR negative	1.11 (1.03–1.18)	1.27 (1.18–1.36)	1.36 (1.21–1.52)	1.39 (1.28–1.52)
Grade 3–4	1.23 (1.17–1.29)	1.34 (1.28–1.41)	1.6 (1.48–1.73)	1.48 (1.39–1.57)
BCS	1.12 (1.02–1.24)	0.96 (0.86–1.07)	1.01 (0.84–1.20)	0.98 (0.86–1.12)
Radiotherapy	1.12 (0.96–1.29)	0.89 (0.83–0.95)	1.31 (1.08–1.6)	0.91 (0.84–0.99)
BCSxRT	0.61 (0.51–0.73)	0.92 (0.8–1.06)	0.63 (0.48–0.82)	0.88 (0.74–1.04)
Year Diagnosis (continuous, year)	0.98 (0.97–0.99)	0.96 (0.95–0.97)	0.92 (0.91–0.94)	0.94 (0.93–0.95)
Age at Diagnosis (continuous, year)	1.05 (1.05–1.05)	1.02 (1.02–1.03)	1.00 (1.00–1.01)	1.00 (1.00–1.01)
Tumor size (continuous, mm)	1.03 (1.03–1.03)	1.02 (1.02–1.02)	1.05 (1.04–1.05)	1.02 (1.02–1.03)
Number positive nodes (continuous, n)	(-)	1.07 (1.07–1.08)	(-)	1.09 (1.08–1.09)
Number nodes examined (continuous, n)	0.99 (0.99–0.99)	0.98 (0.97–0.98)	0.99 (0.99–1.00)	0.97 (0.96–0.97)

BCS = breast conserving surgery, BCSxRT = breast conserving surgery and radiotherapy, ER = estrogen receptor, N0 = node negative, N+ = node positive, PR = progesterone receptor, SEER = Surveillance, Epidemiology, and End Results Program.

**Table 3:** Results of GAM and proportional hazards tests for SEER data

**Overall mortality**	**Node-negative**			**Node-positive**		
		
	no transform	transform age+|age-50|^1.5^	transform age+|age-50|^1.8^	no transform	transform age+|age-50|^1.5^	transform age+|age-50|^1.8^
GAM Chisq for Age (smaller value better)	582.4	6.53	52.4	439.2	18.4	1.83
GAM p-value for Age (larger better)	<.0001	0.089	<.0001	<.0001	0.0003	0.557

Test PH for Age: rho (smaller absolute value better)	0.069	0.071	0.075	0.048	0.044	0.045
Test PH for Age: chisq (smaller value better)	53.6	46.3	48.2	21.1	15.8	16.0

Full PH model Rsquare (larger value better)	0.088	0.094	0.093	0.126	0.135	0.135
Full PH model Likelihood ratio test (larger value better)	5364	5745	5656	3443	3713	3709

**Breast cancer specific mortality**	**Node-negative**			**Node-positive**		
		
	no transform	transform age+|age-50|^1.5^	transform age+|age-50|^1.8^	no transform	transform age+|age-50|^1.5^	transform age+|age-50|^1.8^

GAM Chisq for Age (smaller value better)	52.7	11.1	8.10	63.7	9.20	4.92
GAM p-value for Age (larger better)	<.0001	0.010	0.040	<.0001	0.027	0.179

Test PH for Age: rho (smaller absolute value better)	-0.027	-0.038	-0.042	-0.007	-0.023	-0.027
Test PH for Age: chisq (smaller value better)	2.5	4.5	5.5	0.3	2.8	3.6

Full PH model Rsquare (larger value better)	0.03	0.03	0.031	0.1	0.1	0.1
Full PH model Likelihood ratio test (larger value better)	1779	1799	1803	2698	2715	2716

Chisq = chi-square value, GAM = generalized additive model, PH = proportional hazards. The additional file 3 "output4rev.doc" lists the output of the full PH models.

**Table 4:** Results of GAM and proportional hazards tests for GBSG-2 data

**GBSG-2 data set**	no transform	transform age+|age-50|^1.5^	transform age+|age-50|^1.8^
Hazard ratio for Age	1.001	1.004	1.002
p-value of Hazard ratio for Age	0.92	0.019	0.010

GAM Chisq for Age	31.74	9.29	8.70
GAM p-value for Age	0.0000006	0.0256	0.0336

Test PH for Age: rho	0.131	0.0459	0.0240
Test PH for Age: chisq	6.118	0.581	0.169

Full PH model Rsquare	0.135	0.142	0.143
Full PH model Likelihood ratio test	99.7	105	106

Chisq = chi-square value, GAM = generalized additive model, GBSG-2 = German Breast Cancer Study Group, PH = proportional hazards. The Additional file 2 "outputgbsg2.doc" lists the other covariates included in the PH models.
