# Supplementary material for: Modeling the effect of age in T1-2 breast cancer using the SEER database
Source: BMC Cancer. 2005 Oct 8;5:130. doi: 10.1186/1471-2407-5-130 (PMC1277821; doi:10.1186/1471-2407-5-130)
Supplement: Additional File 1 — Check of the Cox proportional hazards assumption. [file 1471-2407-5-130-S1.doc]

Supplemental Table 2b. Check of the Cox proportional hazards assumption.

| Overall mortality | N0, no age transform | | |  | N0, with age transform | | |  | N+, no age transform | | |  | N+, with age transform | | |
| --- | --- | --- | --- | --- | --- | --- | --- | --- | --- | --- | --- | --- | --- | --- | --- |
|  | rho | chisq | P |  | rho | chisq | P |  | rho | chisq | P |  | rho | chisq | P |
| SEER Central | -0.023 | 4.895 | 0.027 |  | -0.022 | 4.687 | 0.030 |  | -0.002 | 0.028 | 0.867 |  | -0.002 | 0.030 | 0.862 |
| SEER West | -0.004 | 0.178 | 0.673 |  | -0.005 | 0.205 | 0.651 |  | -0.012 | 1.199 | 0.274 |  | -0.012 | 1.050 | 0.306 |
| Race black | -0.035 | 11.481 | 0.001 |  | -0.038 | 13.096 | 0.000 |  | -0.011 | 0.890 | 0.346 |  | -0.012 | 1.158 | 0.282 |
| Married status | 0.010 | 0.958 | 0.328 |  | 0.008 | 0.678 | 0.410 |  | -0.021 | 3.437 | 0.064 |  | -0.021 | 3.591 | 0.058 |
| Inner quadrant | -0.009 | 0.754 | 0.385 |  | -0.009 | 0.826 | 0.363 |  | 0.001 | 0.009 | 0.925 |  | 0.000 | 0.000 | 0.998 |
| Ductal histo. | -0.036 | 11.669 | 0.001 |  | -0.036 | 11.773 | 0.001 |  | -0.020 | 3.195 | 0.074 |  | -0.020 | 3.138 | 0.077 |
| ER negative | -0.014 | 1.695 | 0.193 |  | -0.016 | 2.284 | 0.131 |  | -0.052 | 20.794 | 0.000 |  | -0.053 | 22.066 | 0.000 |
| PR negative | -0.011 | 1.101 | 0.294 |  | -0.011 | 1.099 | 0.294 |  | -0.014 | 1.607 | 0.205 |  | -0.016 | 1.888 | 0.169 |
| Grade 3-4 | -0.052 | 23.981 | 0.000 |  | -0.053 | 25.510 | 0.000 |  | -0.059 | 27.145 | 0.000 |  | -0.064 | 31.190 | 0.000 |
| BCS | -0.031 | 8.934 | 0.003 |  | -0.032 | 9.442 | 0.002 |  | -0.026 | 5.299 | 0.021 |  | -0.030 | 6.922 | 0.009 |
| Radiation | -0.026 | 6.272 | 0.012 |  | -0.026 | 6.257 | 0.012 |  | 0.030 | 7.336 | 0.007 |  | 0.030 | 7.273 | 0.007 |
| BCS*RT | 0.046 | 19.746 | 0.000 |  | 0.046 | 19.275 | 0.000 |  | 0.020 | 3.052 | 0.081 |  | 0.022 | 3.734 | 0.053 |
| Year | 0.005 | 0.191 | 0.662 |  | 0.005 | 0.225 | 0.636 |  | 0.023 | 4.268 | 0.039 |  | 0.023 | 4.226 | 0.040 |
| Age | 0.069 | 53.603 | 0.000 |  | 0.071 | 46.265 | 0.000 |  | 0.048 | 21.056 | 0.000 |  | 0.045 | 15.956 | 0.000 |
| Size | -0.077 | 49.263 | 0.000 |  | -0.077 | 50.464 | 0.000 |  | -0.057 | 23.797 | 0.000 |  | -0.059 | 24.875 | 0.000 |
| Npos | – | – | – |  | – | – | – |  | -0.041 | 13.339 | 0.000 |  | -0.039 | 11.779 | 0.001 |
| Nex | 0.017 | 2.740 | 0.098 |  | 0.014 | 2.059 | 0.151 |  | 0.033 | 9.957 | 0.002 |  | 0.031 | 8.683 | 0.003 |
| GLOBAL | – | 257.542 | 0.000 |  | – | 250.937 | 0.000 |  | – | 226.018 | 0.000 |  | – | 225.386 | 0.000 |
|  |  |  |  |  |  |  |  |  |  |  |  |  |  |  |  |
| Breast cancer specific mortality | N0, no age transform | | |  | N0, with age transform | | |  | N+, no age transform | | |  | N+, with age transform | | |
|  | rho | chisq | P |  | rho | chisq | P |  | rho | chisq | P |  | rho | chisq | P |
| SEER Central | -0.043 | 5.794 | 0.016 |  | -0.042 | 5.474 | 0.019 |  | -0.002 | 0.019 | 0.891 |  | -0.001 | 0.010 | 0.921 |
| SEER West | -0.017 | 0.885 | 0.347 |  | -0.017 | 0.897 | 0.344 |  | -0.015 | 1.219 | 0.270 |  | -0.016 | 1.269 | 0.260 |
| Race black | -0.047 | 6.992 | 0.008 |  | -0.048 | 7.178 | 0.007 |  | -0.008 | 0.357 | 0.550 |  | -0.010 | 0.538 | 0.463 |
| Married status | 0.013 | 0.520 | 0.471 |  | 0.008 | 0.218 | 0.641 |  | -0.013 | 0.813 | 0.367 |  | -0.019 | 1.803 | 0.179 |
| Inner quadrant | -0.033 | 3.302 | 0.069 |  | -0.033 | 3.366 | 0.067 |  | 0.013 | 0.915 | 0.339 |  | 0.013 | 0.795 | 0.373 |
| Ductal histo. | -0.030 | 2.660 | 0.103 |  | -0.030 | 2.769 | 0.096 |  | -0.029 | 4.358 | 0.037 |  | -0.029 | 4.334 | 0.037 |
| ER negative | -0.038 | 4.568 | 0.033 |  | -0.039 | 4.765 | 0.029 |  | -0.079 | 33.764 | 0.000 |  | -0.081 | 35.036 | 0.000 |
| PR negative | -0.044 | 5.758 | 0.016 |  | -0.044 | 5.752 | 0.016 |  | -0.012 | 0.738 | 0.390 |  | -0.011 | 0.701 | 0.403 |
| Grade 3-4 | -0.110 | 38.120 | 0.000 |  | -0.111 | 38.445 | 0.000 |  | -0.072 | 26.776 | 0.000 |  | -0.073 | 27.500 | 0.000 |
| BCS | -0.037 | 4.196 | 0.041 |  | -0.037 | 4.237 | 0.040 |  | -0.030 | 4.644 | 0.031 |  | -0.032 | 5.039 | 0.025 |
| Radiation | -0.044 | 6.003 | 0.014 |  | -0.045 | 6.160 | 0.013 |  | 0.020 | 2.088 | 0.148 |  | 0.018 | 1.695 | 0.193 |
| BCS*RT | 0.049 | 7.282 | 0.007 |  | 0.049 | 7.238 | 0.007 |  | 0.021 | 2.326 | 0.127 |  | 0.022 | 2.492 | 0.114 |
| Year | 0.012 | 0.450 | 0.502 |  | 0.012 | 0.435 | 0.510 |  | 0.020 | 1.993 | 0.158 |  | 0.020 | 1.976 | 0.160 |
| Age | -0.027 | 2.516 | 0.113 |  | -0.038 | 4.516 | 0.034 |  | -0.007 | 0.298 | 0.585 |  | -0.027 | 3.647 | 0.056 |
| Size | -0.079 | 16.106 | 0.000 |  | -0.079 | 15.798 | 0.000 |  | -0.065 | 20.001 | 0.000 |  | -0.064 | 19.570 | 0.000 |
| Npos | – | – | – |  | – | – | – |  | -0.034 | 6.031 | 0.014 |  | -0.033 | 5.470 | 0.019 |
| Nex | 0.002 | 0.017 | 0.896 |  | 0.000 | 0.001 | 0.981 |  | 0.035 | 7.405 | 0.007 |  | 0.033 | 6.542 | 0.011 |
| GLOBAL | – | 168.488 | 0.000 |  | – | 171.105 | 0.000 |  | – | 186.797 | 0.000 |  | – | 190.152 | 0.000 |

Age = for age at diagnosis, age transform = **age**+ **|age–50|^1. 5^** for node negative, or **age**+ **|age–50|^1. 8^** for node positive, BCS = breast conserving surgery, BCS*RT = breast conserving surgery and radiotherapy, Chisq = chi-square value, ER = estrogen receptor, N0 = node negative, N+ = node positive, Npos = number of positive nodes, Nex = nodes examined, PR = progesterone receptor, rho = slope, SEER = Surveillance, Epidemiology, and End Results Program, Size = for tumor size.
