# Supplementary material for: Modeling the effect of age in T1-2 breast cancer using the SEER database
Source: BMC Cancer. 2005 Oct 8;5:130. doi: 10.1186/1471-2407-5-130 (PMC1277821; doi:10.1186/1471-2407-5-130)
Supplement: Additional File 3 — Output of Cox, GAM and cox zph. [file 1471-2407-5-130-S3.doc]

For each combination of

- SEER dataset {N0, N+}
- outcome {overall mortality, breast cancer specific mortality}
- age-model {no transform, transform |age-50|^1.5^, transform |age-50|^1.8^}

do: Cox, GAM, cox.zph.

### Overall mortality

### N0, no age transform

*** Cox Proportional Hazards ***

Call:

coxph(formula = Surv(futime, event) ~ seercent + seerwest + raceblck + married + innerkwd + histduct +

erneg + prneg + g34 + bcs + rt + bcrt + YearDgc + AgeDgc + EodSize + npos + nex, data = m,

na.action = na.exclude, method = "efron")

n= 58139

coef exp(coef) se(coef) z p

seercent -0.00309 0.997 0.027008 -0.115 9.1e-001

seerwest -0.08404 0.919 0.024795 -3.389 7.0e-004

raceblck 0.32102 1.379 0.040848 7.859 3.9e-015

married -0.29203 0.747 0.022146 -13.186 0.0e+000

innerkwd 0.08883 1.093 0.027716 3.205 1.4e-003

histduct 0.13411 1.144 0.024821 5.403 6.5e-008

erneg 0.32734 1.387 0.039476 8.292 1.1e-016

prneg 0.10064 1.106 0.034614 2.908 3.6e-003

g34 0.20340 1.226 0.024460 8.316 1.1e-016

bcs 0.11505 1.122 0.049442 2.327 2.0e-002

rt 0.10871 1.115 0.074339 1.462 1.4e-001

bcrt -0.48767 0.614 0.090887 -5.366 8.1e-008

YearDgc -0.01969 0.980 0.005066 -3.888 1.0e-004

AgeDgc 0.04830 1.049 0.000956 50.542 0.0e+000

EodSize 0.02665 1.027 0.001035 25.747 0.0e+000

npos NA NA 0.000000 NA NA

nex -0.00910 0.991 0.001691 -5.382 7.4e-008

exp(coef) exp(-coef) lower .95 upper .95

seercent 0.997 1.003 0.946 1.051

seerwest 0.919 1.088 0.876 0.965

raceblck 1.379 0.725 1.272 1.493

married 0.747 1.339 0.715 0.780

innerkwd 1.093 0.915 1.035 1.154

histduct 1.144 0.874 1.089 1.201

erneg 1.387 0.721 1.284 1.499

prneg 1.106 0.904 1.033 1.184

g34 1.226 0.816 1.168 1.286

bcs 1.122 0.891 1.018 1.236

rt 1.115 0.897 0.964 1.290

bcrt 0.614 1.629 0.514 0.734

YearDgc 0.980 1.020 0.971 0.990

AgeDgc 1.049 0.953 1.048 1.051

EodSize 1.027 0.974 1.025 1.029

npos NA NA NA NA

nex 0.991 1.009 0.988 0.994

Rsquare= 0.088 (max possible= 0.963 )

Likelihood ratio test= 5364 on 16 df, p=0

Wald test = 5255 on 16 df, p=0

Score (logrank) test = 5486 on 16 df, p=0

*** Generalized Additive Model ***

Call: gam(formula = count ~ s(AgeDgc) + s(EodSize) + s(nex) + s(YearDgc) + offset(log(newtime)), family =

poisson, data = m, na.action = na.exclude)

Deviance Residuals:

Min 1Q Median 3Q Max

-2.585167 -0.5796522 -0.4010854 -0.2279281 3.921486

(Dispersion Parameter for Poisson family taken to be 1 )

Null Deviance: 48965.71 on 58138 degrees of freedom

Residual Deviance: 43711.9 on 58121.99 degrees of freedom

Number of Local Scoring Iterations: 5

DF for Terms and Chi-squares for Nonparametric Effects

Df Npar Df Npar Chisq P(Chi)

(Intercept) 1

s(AgeDgc) 1 3 582.3768 0.00000000

s(EodSize) 1 3 69.9984 0.00000000

s(nex) 1 3 12.1075 0.00695936

s(YearDgc) 1 3 6.7030 0.08239606

> cox.zph(fit2,transform='identity')

rho chisq p

seercent -0.02291 4.895 2.69e-002

seerwest -0.00436 0.178 6.73e-001

raceblck -0.03533 11.481 7.03e-004

married 0.01017 0.958 3.28e-001

innerkwd -0.00902 0.754 3.85e-001

histduct -0.03552 11.669 6.36e-004

erneg -0.01370 1.695 1.93e-001

prneg -0.01101 1.101 2.94e-001

g34 -0.05158 23.981 9.73e-007

bcs -0.03106 8.934 2.80e-003

rt -0.02605 6.272 1.23e-002

bcrt 0.04613 19.746 8.84e-006

YearDgc 0.00458 0.191 6.62e-001

AgeDgc 0.06873 53.603 2.45e-013

EodSize -0.07667 49.263 2.24e-012

npos NA NA NA

nex 0.01661 2.740 9.79e-002

GLOBAL NA 257.542 0.00e+000

### Overall mortality

### N0, m$agetr <- m$AgeDgc+(abs(m$AgeDgc-50))^1.5

*** Cox Proportional Hazards ***

Call:

coxph(formula = Surv(futime, event) ~ seercent + seerwest + raceblck + married + innerkwd + histduct +

erneg + prneg + g34 + bcs + rt + bcrt + YearDgc + agetr + EodSize + npos + nex, data = m,

na.action = na.exclude, method = "efron")

n= 58139

coef exp(coef) se(coef) z p

seercent -0.02740 0.973 0.027027 -1.01 3.1e-001

seerwest -0.09454 0.910 0.024800 -3.81 1.4e-004

raceblck 0.30327 1.354 0.040857 7.42 1.1e-013

married -0.22433 0.799 0.022583 -9.93 0.0e+000

innerkwd 0.09142 1.096 0.027718 3.30 9.7e-004

histduct 0.13576 1.145 0.024814 5.47 4.5e-008

erneg 0.28390 1.328 0.039422 7.20 6.0e-013

prneg 0.11359 1.120 0.034567 3.29 1.0e-003

g34 0.18061 1.198 0.024478 7.38 1.6e-013

bcs 0.07161 1.074 0.049395 1.45 1.5e-001

rt 0.12937 1.138 0.074363 1.74 8.2e-002

bcrt -0.45114 0.637 0.090854 -4.97 6.8e-007

YearDgc -0.01823 0.982 0.005063 -3.60 3.2e-004

agetr 0.00852 1.009 0.000146 58.25 0.0e+000

EodSize 0.02477 1.025 0.001041 23.80 0.0e+000

npos NA NA 0.000000 NA NA

nex -0.00747 0.993 0.001685 -4.43 9.3e-006

exp(coef) exp(-coef) lower .95 upper .95

seercent 0.973 1.028 0.923 1.026

seerwest 0.910 1.099 0.867 0.955

raceblck 1.354 0.738 1.250 1.467

married 0.799 1.251 0.764 0.835

innerkwd 1.096 0.913 1.038 1.157

histduct 1.145 0.873 1.091 1.202

erneg 1.328 0.753 1.230 1.435

prneg 1.120 0.893 1.047 1.199

g34 1.198 0.835 1.142 1.257

bcs 1.074 0.931 0.975 1.183

rt 1.138 0.879 0.984 1.317

bcrt 0.637 1.570 0.533 0.761

YearDgc 0.982 1.018 0.972 0.992

agetr 1.009 0.992 1.008 1.009

EodSize 1.025 0.976 1.023 1.027

npos NA NA NA NA

nex 0.993 1.007 0.989 0.996

Rsquare= 0.094 (max possible= 0.963 )

Likelihood ratio test= 5745 on 16 df, p=0

Wald test = 6332 on 16 df, p=0

Score (logrank) test = 6713 on 16 df, p=0

*** Generalized Additive Model ***

Call: gam(formula = count ~ s(agetr) + s(EodSize) + s(nex) + s(YearDgc) + offset(log(newtime)), family =

poisson, data = m, na.action = na.exclude)

Deviance Residuals:

Min 1Q Median 3Q Max

-2.53996 -0.5805279 -0.3991145 -0.2261915 3.928316

(Dispersion Parameter for Poisson family taken to be 1 )

Null Deviance: 49118.5 on 58138 degrees of freedom

Residual Deviance: 43865.11 on 58121.99 degrees of freedom

Number of Local Scoring Iterations: 5

DF for Terms and Chi-squares for Nonparametric Effects

Df Npar Df Npar Chisq P(Chi)

(Intercept) 1

s(agetr) 1 3 6.52954 0.08892818

s(EodSize) 1 3 68.46733 0.00000000

s(nex) 1 3 11.76885 0.00814384

s(YearDgc) 1 3 6.55601 0.08789645

> cox.zph(fit2,transform='identity')

rho chisq p

seercent -0.02244 4.687 3.04e-002

seerwest -0.00468 0.205 6.51e-001

raceblck -0.03761 13.096 2.96e-004

married 0.00849 0.678 4.10e-001

innerkwd -0.00944 0.826 3.63e-001

histduct -0.03568 11.773 6.01e-004

erneg -0.01589 2.284 1.31e-001

prneg -0.01101 1.099 2.94e-001

g34 -0.05300 25.510 4.40e-007

bcs -0.03195 9.442 2.12e-003

rt -0.02601 6.257 1.24e-002

bcrt 0.04559 19.275 1.13e-005

YearDgc 0.00497 0.225 6.36e-001

agetr 0.07145 46.265 1.03e-011

EodSize -0.07702 50.464 1.21e-012

npos NA NA NA

nex 0.01447 2.059 1.51e-001

GLOBAL NA 250.937 0.00e+000

### Overall mortality

### N0, m$agetr <- m$AgeDgc+(abs(m$AgeDgc-50))^1.8

*** Cox Proportional Hazards ***

Call:

coxph(formula = Surv(futime, event) ~ seercent + seerwest + raceblck + married + innerkwd + histduct +

erneg + prneg + g34 + bcs + rt + bcrt + YearDgc + agetr + EodSize + npos + nex, data = m,

na.action = na.exclude, method = "efron")

n= 58139

coef exp(coef) se(coef) z p

seercent -0.02915 0.971 0.0270316 -1.08 2.8e-001

seerwest -0.09710 0.907 0.0248008 -3.92 9.0e-005

raceblck 0.28684 1.332 0.0408520 7.02 2.2e-012

married -0.23020 0.794 0.0226690 -10.15 0.0e+000

innerkwd 0.09241 1.097 0.0277179 3.33 8.6e-004

histduct 0.13426 1.144 0.0248115 5.41 6.3e-008

erneg 0.25867 1.295 0.0393945 6.57 5.2e-011

prneg 0.12153 1.129 0.0345590 3.52 4.4e-004

g34 0.16968 1.185 0.0244836 6.93 4.2e-012

bcs 0.05336 1.055 0.0493812 1.08 2.8e-001

rt 0.12182 1.130 0.0743646 1.64 1.0e-001

bcrt -0.43819 0.645 0.0908465 -4.82 1.4e-006

YearDgc -0.01784 0.982 0.0050606 -3.53 4.2e-004

agetr 0.00310 1.003 0.0000525 59.02 0.0e+000

EodSize 0.02406 1.024 0.0010429 23.07 0.0e+000

npos NA NA 0.0000000 NA NA

nex -0.00755 0.992 0.0016846 -4.48 7.4e-006

exp(coef) exp(-coef) lower .95 upper .95

seercent 0.971 1.030 0.921 1.024

seerwest 0.907 1.102 0.864 0.953

raceblck 1.332 0.751 1.230 1.443

married 0.794 1.259 0.760 0.830

innerkwd 1.097 0.912 1.039 1.158

histduct 1.144 0.874 1.089 1.201

erneg 1.295 0.772 1.199 1.399

prneg 1.129 0.886 1.055 1.208

g34 1.185 0.844 1.129 1.243

bcs 1.055 0.948 0.958 1.162

rt 1.130 0.885 0.976 1.307

bcrt 0.645 1.550 0.540 0.771

YearDgc 0.982 1.018 0.973 0.992

agetr 1.003 0.997 1.003 1.003

EodSize 1.024 0.976 1.022 1.026

npos NA NA NA NA

nex 0.992 1.008 0.989 0.996

Rsquare= 0.093 (max possible= 0.963 )

Likelihood ratio test= 5656 on 16 df, p=0

Wald test = 6558 on 16 df, p=0

Score (logrank) test = 6962 on 16 df, p=0

*** Generalized Additive Model ***

Call: gam(formula = count ~ s(agetr) + s(EodSize) + s(nex) + s(YearDgc) + offset(log(newtime)), family =

poisson, data = m, na.action = na.exclude)

Deviance Residuals:

Min 1Q Median 3Q Max

-2.514697 -0.5803435 -0.4003571 -0.2269407 3.936066

(Dispersion Parameter for Poisson family taken to be 1 )

Null Deviance: 49145.74 on 58138 degrees of freedom

Residual Deviance: 43922.54 on 58121.99 degrees of freedom

Number of Local Scoring Iterations: 5

DF for Terms and Chi-squares for Nonparametric Effects

Df Npar Df Npar Chisq P(Chi)

(Intercept) 1

s(agetr) 1 3 52.36234 0.00000000

s(EodSize) 1 3 66.54938 0.00000000

s(nex) 1 3 11.62237 0.00871601

s(YearDgc) 1 3 6.36308 0.09567451

> cox.zph(fit2,transform='identity')

rho chisq p

seercent -0.02213 4.558 3.28e-002

seerwest -0.00424 0.168 6.82e-001

raceblck -0.03836 13.641 2.21e-004

married 0.00752 0.535 4.64e-001

innerkwd -0.00949 0.834 3.61e-001

histduct -0.03543 11.602 6.59e-004

erneg -0.01642 2.440 1.18e-001

prneg -0.01112 1.121 2.90e-001

g34 -0.05326 25.815 3.76e-007

bcs -0.03198 9.456 2.10e-003

rt -0.02626 6.380 1.15e-002

bcrt 0.04555 19.239 1.15e-005

YearDgc 0.00540 0.265 6.07e-001

agetr 0.07477 48.189 3.87e-012

EodSize -0.07763 51.541 7.01e-013

npos NA NA NA

nex 0.01412 1.960 1.62e-001

GLOBAL NA 253.876 0.00e+000

### Overall mortality

### N+, no age transform

*** Cox Proportional Hazards ***

Call:

coxph(formula = Surv(futime, event) ~ seercent + seerwest + raceblck + married + innerkwd + histduct +

erneg + prneg + g34 + bcs + rt + bcrt + YearDgc + AgeDgc + EodSize + npos + nex, data = m,

na.action = na.exclude, method = "efron")

n= 25665

coef exp(coef) se(coef) z p

seercent -0.0777 0.925 0.029734 -2.614 9.0e-003

seerwest -0.1419 0.868 0.027254 -5.207 1.9e-007

raceblck 0.3469 1.415 0.037823 9.172 0.0e+000

married -0.1975 0.821 0.023857 -8.279 1.1e-016

innerkwd 0.1457 1.157 0.035837 4.064 4.8e-005

histduct 0.0996 1.105 0.028783 3.461 5.4e-004

erneg 0.4192 1.521 0.039257 10.677 0.0e+000

prneg 0.2366 1.267 0.036220 6.532 6.5e-011

g34 0.2934 1.341 0.024233 12.109 0.0e+000

bcs -0.0407 0.960 0.056920 -0.715 4.7e-001

rt -0.1206 0.886 0.036261 -3.325 8.9e-004

bcrt -0.0833 0.920 0.071758 -1.161 2.5e-001

YearDgc -0.0457 0.955 0.005228 -8.734 0.0e+000

AgeDgc 0.0239 1.024 0.000893 26.770 0.0e+000

EodSize 0.0202 1.020 0.001044 19.366 0.0e+000

npos 0.0681 1.070 0.002153 31.635 0.0e+000

nex -0.0255 0.975 0.001931 -13.214 0.0e+000

exp(coef) exp(-coef) lower .95 upper .95

seercent 0.925 1.081 0.873 0.981

seerwest 0.868 1.152 0.823 0.915

raceblck 1.415 0.707 1.314 1.524

married 0.821 1.218 0.783 0.860

innerkwd 1.157 0.864 1.078 1.241

histduct 1.105 0.905 1.044 1.169

erneg 1.521 0.658 1.408 1.642

prneg 1.267 0.789 1.180 1.360

g34 1.341 0.746 1.279 1.406

bcs 0.960 1.042 0.859 1.073

rt 0.886 1.128 0.826 0.952

bcrt 0.920 1.087 0.799 1.059

YearDgc 0.955 1.047 0.946 0.965

AgeDgc 1.024 0.976 1.022 1.026

EodSize 1.020 0.980 1.018 1.023

npos 1.070 0.934 1.066 1.075

nex 0.975 1.026 0.971 0.979

Rsquare= 0.126 (max possible= 0.997 )

Likelihood ratio test= 3443 on 17 df, p=0

Wald test = 3664 on 17 df, p=0

Score (logrank) test = 3800 on 17 df, p=0

*** Generalized Additive Model ***

Call: gam(formula = count ~ s(AgeDgc) + s(EodSize) + s(npos) + s(nex) + s(YearDgc) + offset(log(newtime)),

family = poisson, data = m, na.action = na.exclude)

Deviance Residuals:

Min 1Q Median 3Q Max

-2.782689 -0.8006689 -0.5325092 0.6608838 3.935739

(Dispersion Parameter for Poisson family taken to be 1 )

Null Deviance: 31560.35 on 25664 degrees of freedom

Residual Deviance: 28495.2 on 25645.5 degrees of freedom

Number of Local Scoring Iterations: 4

DF for Terms and Chi-squares for Nonparametric Effects

Df Npar Df Npar Chisq P(Chi)

(Intercept) 1

s(AgeDgc) 1 2.7 439.1919 0.00000000

s(EodSize) 1 2.7 76.6029 0.00000000

s(npos) 1 2.7 103.7585 0.00000000

s(nex) 1 2.7 37.1970 0.00000003

s(YearDgc) 1 2.7 6.1852 0.08439732

> cox.zph(fit2,transform='identity')

rho chisq p

seercent -0.00189 0.02814 8.67e-001

seerwest -0.01235 1.19885 2.74e-001

raceblck -0.01071 0.88963 3.46e-001

married -0.02117 3.43740 6.37e-002

innerkwd 0.00106 0.00878 9.25e-001

histduct -0.02020 3.19469 7.39e-002

erneg -0.05152 20.79359 5.12e-006

prneg -0.01426 1.60700 2.05e-001

g34 -0.05937 27.14474 1.89e-007

bcs -0.02607 5.29883 2.13e-002

rt 0.03032 7.33598 6.76e-003

bcrt 0.01977 3.05232 8.06e-002

YearDgc 0.02349 4.26773 3.88e-002

AgeDgc 0.04798 21.05639 4.46e-006

EodSize -0.05747 23.79686 1.07e-006

npos -0.04147 13.33925 2.60e-004

nex 0.03308 9.95684 1.60e-003

GLOBAL NA 226.01795 0.00e+000

### Overall mortality

### N+, m$agetr <- m$AgeDgc+(abs(m$AgeDgc-50))^1.5

*** Cox Proportional Hazards ***

Call:

coxph(formula = Surv(futime, event) ~ seercent + seerwest + raceblck + married + innerkwd + histduct +

erneg + prneg + g34 + bcs + rt + bcrt + YearDgc + agetr + EodSize + npos + nex, data = m,

na.action = na.exclude, method = "efron")

n= 25665

coef exp(coef) se(coef) z p

seercent -0.08574 0.918 0.029739 -2.883 3.9e-003

seerwest -0.13745 0.872 0.027248 -5.045 4.5e-007

raceblck 0.35381 1.424 0.037834 9.352 0.0e+000

married -0.13198 0.876 0.024357 -5.419 6.0e-008

innerkwd 0.15375 1.166 0.035835 4.291 1.8e-005

histduct 0.09635 1.101 0.028766 3.350 8.1e-004

erneg 0.41097 1.508 0.039110 10.508 0.0e+000

prneg 0.23426 1.264 0.036101 6.489 8.6e-011

g34 0.27770 1.320 0.024220 11.466 0.0e+000

bcs -0.05517 0.946 0.056805 -0.971 3.3e-001

rt -0.08974 0.914 0.036351 -2.469 1.4e-002

bcrt -0.07357 0.929 0.071716 -1.026 3.0e-001

YearDgc -0.04534 0.956 0.005230 -8.669 0.0e+000

agetr 0.00546 1.005 0.000166 32.891 0.0e+000

EodSize 0.01926 1.019 0.001043 18.470 0.0e+000

npos 0.06810 1.070 0.002148 31.700 0.0e+000

nex -0.02481 0.975 0.001927 -12.877 0.0e+000

exp(coef) exp(-coef) lower .95 upper .95

seercent 0.918 1.090 0.866 0.973

seerwest 0.872 1.147 0.826 0.919

raceblck 1.424 0.702 1.323 1.534

married 0.876 1.141 0.836 0.919

innerkwd 1.166 0.857 1.087 1.251

histduct 1.101 0.908 1.041 1.165

erneg 1.508 0.663 1.397 1.628

prneg 1.264 0.791 1.178 1.357

g34 1.320 0.758 1.259 1.384

bcs 0.946 1.057 0.847 1.058

rt 0.914 1.094 0.851 0.982

bcrt 0.929 1.076 0.807 1.069

YearDgc 0.956 1.046 0.946 0.966

agetr 1.005 0.995 1.005 1.006

EodSize 1.019 0.981 1.017 1.022

npos 1.070 0.934 1.066 1.075

nex 0.975 1.025 0.972 0.979

Rsquare= 0.135 (max possible= 0.997 )

Likelihood ratio test= 3713 on 17 df, p=0

Wald test = 4072 on 17 df, p=0

Score (logrank) test = 4227 on 17 df, p=0

*** Generalized Additive Model ***

Call: gam(formula = count ~ s(agetr) + s(EodSize) + s(npos) + s(nex) + s(YearDgc) + offset(log(newtime)),

family = poisson, data = m, na.action = na.exclude)

Deviance Residuals:

Min 1Q Median 3Q Max

-2.870861 -0.7996499 -0.5303153 0.6610839 3.946443

(Dispersion Parameter for Poisson family taken to be 1 )

Null Deviance: 31658.92 on 25664 degrees of freedom

Residual Deviance: 28594.98 on 25645.46 degrees of freedom

Number of Local Scoring Iterations: 4

DF for Terms and Chi-squares for Nonparametric Effects

Df Npar Df Npar Chisq P(Chi)

(Intercept) 1

s(agetr) 1 2.7 18.4476 0.00025864

s(EodSize) 1 2.7 76.5467 0.00000000

s(npos) 1 2.7 102.9324 0.00000000

s(nex) 1 2.7 36.3901 0.00000004

s(YearDgc) 1 2.7 6.1295 0.08636529

> cox.zph(fit2,transform='identity')

rho chisq p

seercent -0.0021166 3.51e-002 8.51e-001

seerwest -0.0118383 1.10e+000 2.94e-001

raceblck -0.0122266 1.17e+000 2.80e-001

married -0.0208994 3.40e+000 6.51e-002

innerkwd -0.0000927 6.66e-005 9.93e-001

histduct -0.0200536 3.15e+000 7.62e-002

erneg -0.0527229 2.17e+001 3.12e-006

prneg -0.0150915 1.79e+000 1.81e-001

g34 -0.0629763 3.06e+001 3.15e-008

bcs -0.0289135 6.51e+000 1.07e-002

rt 0.0302696 7.33e+000 6.79e-003

bcrt 0.0214003 3.57e+000 5.87e-002

YearDgc 0.0231298 4.14e+000 4.18e-002

agetr 0.0444026 1.58e+001 7.18e-005

EodSize -0.0580746 2.43e+001 8.37e-007

npos -0.0391863 1.19e+001 5.70e-004

nex 0.0310700 8.77e+000 3.06e-003

GLOBAL NA 2.23e+002 0.00e+000

### Overall mortality

### N+, m$agetr <- m$AgeDgc+(abs(m$AgeDgc-50))^1.8

*** Cox Proportional Hazards ***

Call:

coxph(formula = Surv(futime, event) ~ seercent + seerwest + raceblck + married + innerkwd + histduct +

erneg + prneg + g34 + bcs + rt + bcrt + YearDgc + agetr + EodSize + npos + nex, data = m,

na.action = na.exclude, method = "efron")

n= 25665

coef exp(coef) se(coef) z p

seercent -0.08539 0.918 0.0297428 -2.871 4.1e-003

seerwest -0.13688 0.872 0.0272508 -5.023 5.1e-007

raceblck 0.34897 1.418 0.0378393 9.222 0.0e+000

married -0.13156 0.877 0.0244201 -5.387 7.2e-008

innerkwd 0.15569 1.168 0.0358370 4.345 1.4e-005

histduct 0.09519 1.100 0.0287627 3.310 9.3e-004

erneg 0.40147 1.494 0.0390720 10.275 0.0e+000

prneg 0.23554 1.266 0.0360808 6.528 6.7e-011

g34 0.27245 1.313 0.0242195 11.249 0.0e+000

bcs -0.07159 0.931 0.0567621 -1.261 2.1e-001

rt -0.08917 0.915 0.0363582 -2.453 1.4e-002

bcrt -0.06090 0.941 0.0716929 -0.849 4.0e-001

YearDgc -0.04549 0.956 0.0052284 -8.701 0.0e+000

agetr 0.00208 1.002 0.0000624 33.337 0.0e+000

EodSize 0.01890 1.019 0.0010428 18.124 0.0e+000

npos 0.06829 1.071 0.0021470 31.807 0.0e+000

nex -0.02494 0.975 0.0019262 -12.949 0.0e+000

exp(coef) exp(-coef) lower .95 upper .95

seercent 0.918 1.089 0.866 0.973

seerwest 0.872 1.147 0.827 0.920

raceblck 1.418 0.705 1.316 1.527

married 0.877 1.141 0.836 0.920

innerkwd 1.168 0.856 1.089 1.253

histduct 1.100 0.909 1.040 1.164

erneg 1.494 0.669 1.384 1.613

prneg 1.266 0.790 1.179 1.358

g34 1.313 0.762 1.252 1.377

bcs 0.931 1.074 0.833 1.040

rt 0.915 1.093 0.852 0.982

bcrt 0.941 1.063 0.818 1.083

YearDgc 0.956 1.047 0.946 0.965

agetr 1.002 0.998 1.002 1.002

EodSize 1.019 0.981 1.017 1.021

npos 1.071 0.934 1.066 1.075

nex 0.975 1.025 0.972 0.979

Rsquare= 0.135 (max possible= 0.997 )

Likelihood ratio test= 3709 on 17 df, p=0

Wald test = 4129 on 17 df, p=0

Score (logrank) test = 4288 on 17 df, p=0

*** Generalized Additive Model ***

Call: gam(formula = count ~ s(agetr) + s(EodSize) + s(npos) + s(nex) + s(YearDgc) + offset(log(newtime)),

family = poisson, data = m, na.action = na.exclude)

Deviance Residuals:

Min 1Q Median 3Q Max

-2.877607 -0.8003558 -0.5303699 0.6613224 3.949064

(Dispersion Parameter for Poisson family taken to be 1 )

Null Deviance: 31671.08 on 25664 degrees of freedom

Residual Deviance: 28607.38 on 25645.46 degrees of freedom

Number of Local Scoring Iterations: 4

DF for Terms and Chi-squares for Nonparametric Effects

Df Npar Df Npar Chisq P(Chi)

(Intercept) 1

s(agetr) 1 2.7 1.8332 0.5568318

s(EodSize) 1 2.7 76.4575 0.0000000

s(npos) 1 2.7 102.8031 0.0000000

s(nex) 1 2.7 36.2867 0.0000000

s(YearDgc) 1 2.7 5.8987 0.0958841

> cox.zph(fit2,transform='identity')

rho chisq p

seercent -0.0019657 3.03e-002 8.62e-001

seerwest -0.0115592 1.05e+000 3.06e-001

raceblck -0.0121798 1.16e+000 2.82e-001

married -0.0214013 3.59e+000 5.81e-002

innerkwd -0.0000266 5.49e-006 9.98e-001

histduct -0.0200310 3.14e+000 7.65e-002

erneg -0.0531227 2.21e+001 2.64e-006

prneg -0.0155111 1.89e+000 1.69e-001

g34 -0.0635315 3.12e+001 2.34e-008

bcs -0.0298149 6.92e+000 8.51e-003

rt 0.0301488 7.27e+000 7.00e-003

bcrt 0.0218729 3.73e+000 5.33e-002

YearDgc 0.0233647 4.23e+000 3.98e-002

agetr 0.0451918 1.60e+001 6.48e-005

EodSize -0.0587696 2.49e+001 6.12e-007

npos -0.0390484 1.18e+001 5.99e-004

nex 0.0309276 8.68e+000 3.21e-003

GLOBAL NA 2.25e+002 0.00e+000

### Breast-cancer specific cause of death

### N0, no transform

*** Cox Proportional Hazards ***

Call:

coxph(formula = Surv(futime, event2) ~ seercent + seerwest + raceblck + married + innerkwd +

histduct + erneg + prneg + g34 + bcs + rt + bcrt + YearDgc + AgeDgc + EodSize + npos + nex,

data = m, na.action = na.exclude, method = "efron")

n= 58139

coef exp(coef) se(coef) z p

seercent 0.03261 1.033 0.04700 0.6940 4.9e-001

seerwest -0.19675 0.821 0.04350 -4.5228 6.1e-006

raceblck 0.31679 1.373 0.06303 5.0256 5.0e-007

married -0.13121 0.877 0.03803 -3.4501 5.6e-004

innerkwd 0.27887 1.322 0.04500 6.1977 5.7e-010

histduct 0.36269 1.437 0.04676 7.7563 8.7e-015

erneg 0.45404 1.575 0.06143 7.3912 1.5e-013

prneg 0.30618 1.358 0.05753 5.3225 1.0e-007

g34 0.46763 1.596 0.03961 11.8066 0.0e+000

bcs 0.00686 1.007 0.09105 0.0754 9.4e-001

rt 0.27136 1.312 0.10055 2.6988 7.0e-003

bcrt -0.46530 0.628 0.13807 -3.3700 7.5e-004

YearDgc -0.07912 0.924 0.00878 -9.0119 0.0e+000

AgeDgc 0.00317 1.003 0.00144 2.2025 2.8e-002

EodSize 0.04562 1.047 0.00166 27.4915 0.0e+000

npos NA NA 0.00000 NA NA

nex -0.00839 0.992 0.00287 -2.9285 3.4e-003

exp(coef) exp(-coef) lower .95 upper .95

seercent 1.033 0.968 0.942 1.133

seerwest 0.821 1.217 0.754 0.895

raceblck 1.373 0.728 1.213 1.553

married 0.877 1.140 0.814 0.945

innerkwd 1.322 0.757 1.210 1.443

histduct 1.437 0.696 1.311 1.575

erneg 1.575 0.635 1.396 1.776

prneg 1.358 0.736 1.213 1.520

g34 1.596 0.626 1.477 1.725

bcs 1.007 0.993 0.842 1.204

rt 1.312 0.762 1.077 1.597

bcrt 0.628 1.592 0.479 0.823

YearDgc 0.924 1.082 0.908 0.940

AgeDgc 1.003 0.997 1.000 1.006

EodSize 1.047 0.955 1.043 1.050

npos NA NA NA NA

nex 0.992 1.008 0.986 0.997

Rsquare= 0.03 (max possible= 0.668 )

Likelihood ratio test= 1779 on 16 df, p=0

Wald test = 1988 on 16 df, p=0

Score (logrank) test = 2104 on 16 df, p=0

*** Generalized Additive Model ***

Call: gam(formula = count ~ s(AgeDgc) + s(EodSize) + s(nex) + s(YearDgc) + offset(log(newtime)), family =

poisson, data = m, na.action = na.exclude)

Deviance Residuals:

Min 1Q Median 3Q Max

-0.9801817 -0.3667304 -0.2568122 -0.17266 4.253697

(Dispersion Parameter for Poisson family taken to be 1 )

Null Deviance: 22960.1 on 58138 degrees of freedom

Residual Deviance: 21639.11 on 58122.32 degrees of freedom

Number of Local Scoring Iterations: 5

DF for Terms and Chi-squares for Nonparametric Effects

Df Npar Df Npar Chisq P(Chi)

(Intercept) 1

s(AgeDgc) 1 2.9 52.7182 0.0000000

s(EodSize) 1 2.9 172.2376 0.0000000

s(nex) 1 2.9 2.8650 0.3930693

s(YearDgc) 1 3.0 3.1789 0.3644891

> cox.zph(fit2,transform='identity')

rho chisq p

seercent -0.04332 5.7939 1.61e-002

seerwest -0.01682 0.8846 3.47e-001

raceblck -0.04743 6.9922 8.19e-003

married 0.01304 0.5196 4.71e-001

innerkwd -0.03287 3.3018 6.92e-002

histduct -0.02956 2.6603 1.03e-001

erneg -0.03844 4.5683 3.26e-002

prneg -0.04379 5.7583 1.64e-002

g34 -0.11048 38.1201 6.65e-010

bcs -0.03709 4.1961 4.05e-002

rt -0.04430 6.0026 1.43e-002

bcrt 0.04880 7.2824 6.96e-003

YearDgc 0.01232 0.4498 5.02e-001

AgeDgc -0.02713 2.5162 1.13e-001

EodSize -0.07918 16.1056 5.99e-005

npos NA NA NA

nex 0.00231 0.0171 8.96e-001

GLOBAL NA 168.4884 0.00e+000

### Breast-cancer specific cause of death

### N0, m$agetr <- m$AgeDgc+(abs(m$AgeDgc-50))^1.5

*** Cox Proportional Hazards ***

Call:

coxph(formula = Surv(futime, event2) ~ seercent + seerwest + raceblck + married + innerkwd +

histduct + erneg + prneg + g34 + bcs + rt + bcrt + YearDgc + agetr + EodSize + npos + nex,

data = m, na.action = na.exclude, method = "efron")

n= 58139

coef exp(coef) se(coef) z p

seercent 0.02364 1.024 0.047033 0.503 6.2e-001

seerwest -0.19678 0.821 0.043493 -4.524 6.1e-006

raceblck 0.33157 1.393 0.062913 5.270 1.4e-007

married -0.09612 0.908 0.038619 -2.489 1.3e-002

innerkwd 0.27972 1.323 0.044996 6.217 5.1e-010

histduct 0.36529 1.441 0.046765 7.811 5.7e-015

erneg 0.46850 1.598 0.061158 7.661 1.9e-014

prneg 0.30219 1.353 0.057483 5.257 1.5e-007

g34 0.47311 1.605 0.039498 11.978 0.0e+000

bcs 0.01231 1.012 0.090954 0.135 8.9e-001

rt 0.28880 1.335 0.100574 2.871 4.1e-003

bcrt -0.46870 0.626 0.138034 -3.396 6.8e-004

YearDgc -0.07926 0.924 0.008778 -9.029 0.0e+000

agetr 0.00147 1.001 0.000289 5.089 3.6e-007

EodSize 0.04571 1.047 0.001655 27.627 0.0e+000

npos NA NA 0.000000 NA NA

nex -0.00742 0.993 0.002863 -2.592 9.5e-003

exp(coef) exp(-coef) lower .95 upper .95

seercent 1.024 0.977 0.934 1.123

seerwest 0.821 1.217 0.754 0.894

raceblck 1.393 0.718 1.232 1.576

married 0.908 1.101 0.842 0.980

innerkwd 1.323 0.756 1.211 1.445

histduct 1.441 0.694 1.315 1.579

erneg 1.598 0.626 1.417 1.801

prneg 1.353 0.739 1.209 1.514

g34 1.605 0.623 1.485 1.734

bcs 1.012 0.988 0.847 1.210

rt 1.335 0.749 1.096 1.626

bcrt 0.626 1.598 0.477 0.820

YearDgc 0.924 1.082 0.908 0.940

agetr 1.001 0.999 1.001 1.002

EodSize 1.047 0.955 1.043 1.050

npos NA NA NA NA

nex 0.993 1.007 0.987 0.998

Rsquare= 0.03 (max possible= 0.668 )

Likelihood ratio test= 1799 on 16 df, p=0

Wald test = 2004 on 16 df, p=0

Score (logrank) test = 2122 on 16 df, p=0

*** Generalized Additive Model ***

Call: gam(formula = count ~ s(agetr) + s(EodSize) + s(nex) + s(YearDgc) + offset(log(newtime)), family =

poisson, data = m, na.action = na.exclude)

Deviance Residuals:

Min 1Q Median 3Q Max

-0.8992718 -0.3672661 -0.2576106 -0.172994 4.239983

(Dispersion Parameter for Poisson family taken to be 1 )

Null Deviance: 22970.93 on 58138 degrees of freedom

Residual Deviance: 21677.97 on 58122.34 degrees of freedom

Number of Local Scoring Iterations: 5

DF for Terms and Chi-squares for Nonparametric Effects

Df Npar Df Npar Chisq P(Chi)

(Intercept) 1

s(agetr) 1 2.9 11.0979 0.0101088

s(EodSize) 1 2.9 176.8000 0.0000000

s(nex) 1 2.9 2.8837 0.3909569

s(YearDgc) 1 3.0 3.3036 0.3468009

> cox.zph(fit2,transform='identity')

rho chisq p

seercent -0.042114 5.47e+000 1.93e-002

seerwest -0.016939 8.97e-001 3.44e-001

raceblck -0.047948 7.18e+000 7.38e-003

married 0.008436 2.18e-001 6.41e-001

innerkwd -0.033180 3.37e+000 6.66e-002

histduct -0.030155 2.77e+000 9.61e-002

erneg -0.039385 4.77e+000 2.90e-002

prneg -0.043764 5.75e+000 1.65e-002

g34 -0.110917 3.84e+001 5.63e-010

bcs -0.037287 4.24e+000 3.96e-002

rt -0.044868 6.16e+000 1.31e-002

bcrt 0.048660 7.24e+000 7.14e-003

YearDgc 0.012103 4.35e-001 5.10e-001

agetr -0.037986 4.52e+000 3.36e-002

EodSize -0.078624 1.58e+001 7.05e-005

npos NA NA NA

nex 0.000414 5.47e-004 9.81e-001

GLOBAL NA 1.71e+002 0.00e+000

### Breast-cancer specific cause of death

### N0, m$agetr <- m$AgeDgc+(abs(m$AgeDgc-50))^1.8

*** Cox Proportional Hazards ***

Call:

coxph(formula = Surv(futime, event2) ~ seercent + seerwest + raceblck + married + innerkwd +

histduct + erneg + prneg + g34 + bcs + rt + bcrt + YearDgc + agetr + EodSize + npos + nex,

data = m, na.action = na.exclude, method = "efron")

n= 58139

coef exp(coef) se(coef) z p

seercent 0.022243 1.022 0.047040 0.473 6.4e-001

seerwest -0.197140 0.821 0.043492 -4.533 5.8e-006

raceblck 0.331514 1.393 0.062879 5.272 1.3e-007

married -0.092158 0.912 0.038672 -2.383 1.7e-002

innerkwd 0.279922 1.323 0.044996 6.221 4.9e-010

histduct 0.365521 1.441 0.046765 7.816 5.4e-015

erneg 0.467217 1.596 0.061091 7.648 2.0e-014

prneg 0.302925 1.354 0.057475 5.271 1.4e-007

g34 0.472340 1.604 0.039478 11.965 0.0e+000

bcs 0.010645 1.011 0.090933 0.117 9.1e-001

rt 0.290315 1.337 0.100572 2.887 3.9e-003

bcrt -0.467767 0.626 0.138027 -3.389 7.0e-004

YearDgc -0.079268 0.924 0.008778 -9.030 0.0e+000

agetr 0.000607 1.001 0.000112 5.443 5.2e-008

EodSize 0.045641 1.047 0.001654 27.590 0.0e+000

npos NA NA 0.000000 NA NA

nex -0.007326 0.993 0.002862 -2.560 1.0e-002

exp(coef) exp(-coef) lower .95 upper .95

seercent 1.022 0.978 0.932 1.121

seerwest 0.821 1.218 0.754 0.894

raceblck 1.393 0.718 1.232 1.576

married 0.912 1.097 0.845 0.984

innerkwd 1.323 0.756 1.211 1.445

histduct 1.441 0.694 1.315 1.580

erneg 1.596 0.627 1.415 1.799

prneg 1.354 0.739 1.210 1.515

g34 1.604 0.624 1.484 1.733

bcs 1.011 0.989 0.846 1.208

rt 1.337 0.748 1.098 1.628

bcrt 0.626 1.596 0.478 0.821

YearDgc 0.924 1.082 0.908 0.940

agetr 1.001 0.999 1.000 1.001

EodSize 1.047 0.955 1.043 1.050

npos NA NA NA NA

nex 0.993 1.007 0.987 0.998

Rsquare= 0.031 (max possible= 0.668 )

Likelihood ratio test= 1803 on 16 df, p=0

Wald test = 2007 on 16 df, p=0

Score (logrank) test = 2125 on 16 df, p=0

*** Generalized Additive Model ***

Call: gam(formula = count ~ s(agetr) + s(EodSize) + s(nex) + s(YearDgc) + offset(log(newtime)), family =

poisson, data = m, na.action = na.exclude)

Deviance Residuals:

Min 1Q Median 3Q Max

-0.8984844 -0.367155 -0.2575049 -0.1729315 4.246975

(Dispersion Parameter for Poisson family taken to be 1 )

Null Deviance: 22973.12 on 58138 degrees of freedom

Residual Deviance: 21679.7 on 58122.36 degrees of freedom

Number of Local Scoring Iterations: 5

DF for Terms and Chi-squares for Nonparametric Effects

Df Npar Df Npar Chisq P(Chi)

(Intercept) 1

s(agetr) 1 2.9 8.0978 0.0396197

s(EodSize) 1 2.9 177.0641 0.0000000

s(nex) 1 2.9 2.8798 0.3915187

s(YearDgc) 1 3.0 3.3239 0.3439921

> cox.zph(fit2,transform='identity')

rho chisq p

seercent -0.041744 5.38e+000 2.04e-002

seerwest -0.016888 8.91e-001 3.45e-001

raceblck -0.048191 7.26e+000 7.05e-003

married 0.006938 1.48e-001 7.01e-001

innerkwd -0.033330 3.40e+000 6.54e-002

histduct -0.030221 2.78e+000 9.54e-002

erneg -0.039519 4.79e+000 2.86e-002

prneg -0.043830 5.77e+000 1.63e-002

g34 -0.110917 3.85e+001 5.53e-010

bcs -0.037202 4.22e+000 4.00e-002

rt -0.045123 6.23e+000 1.26e-002

bcrt 0.048616 7.22e+000 7.19e-003

YearDgc 0.012165 4.39e-001 5.08e-001

agetr -0.041904 5.48e+000 1.93e-002

EodSize -0.078268 1.57e+001 7.58e-005

npos NA NA NA

nex -0.000168 9.03e-005 9.92e-001

GLOBAL NA 1.72e+002 0.00e+000

### Breast-cancer specific cause of death

### N+, no transform

*** Cox Proportional Hazards ***

Call:

coxph(formula = Surv(futime, event2) ~ seercent + seerwest + raceblck + married + innerkwd +

histduct + erneg + prneg + g34 + bcs + rt + bcrt + YearDgc + AgeDgc + EodSize + npos + nex,

data = m, na.action = na.exclude, method = "efron")

n= 25665

coef exp(coef) se(coef) z p

seercent -0.08154 0.922 0.03720 -2.192 2.8e-002

seerwest -0.15627 0.855 0.03376 -4.629 3.7e-006

raceblck 0.36115 1.435 0.04527 7.977 1.6e-015

married -0.09379 0.910 0.02978 -3.149 1.6e-003

innerkwd 0.26066 1.298 0.04258 6.122 9.3e-010

histduct 0.11671 1.124 0.03597 3.244 1.2e-003

erneg 0.45811 1.581 0.04657 9.838 0.0e+000

prneg 0.33067 1.392 0.04392 7.529 5.1e-014

g34 0.39110 1.479 0.02998 13.045 0.0e+000

bcs -0.01563 0.984 0.06749 -0.232 8.2e-001

rt -0.09164 0.912 0.04240 -2.161 3.1e-002

bcrt -0.13008 0.878 0.08490 -1.532 1.3e-001

YearDgc -0.06465 0.937 0.00645 -10.030 0.0e+000

AgeDgc 0.00283 1.003 0.00108 2.617 8.9e-003

EodSize 0.02413 1.024 0.00128 18.848 0.0e+000

npos 0.08383 1.087 0.00253 33.174 0.0e+000

nex -0.03427 0.966 0.00242 -14.133 0.0e+000

exp(coef) exp(-coef) lower .95 upper .95

seercent 0.922 1.085 0.857 0.991

seerwest 0.855 1.169 0.801 0.914

raceblck 1.435 0.697 1.313 1.568

married 0.910 1.098 0.859 0.965

innerkwd 1.298 0.771 1.194 1.411

histduct 1.124 0.890 1.047 1.206

erneg 1.581 0.632 1.443 1.732

prneg 1.392 0.718 1.277 1.517

g34 1.479 0.676 1.394 1.568

bcs 0.984 1.016 0.863 1.124

rt 0.912 1.096 0.840 0.992

bcrt 0.878 1.139 0.743 1.037

YearDgc 0.937 1.067 0.926 0.949

AgeDgc 1.003 0.997 1.001 1.005

EodSize 1.024 0.976 1.022 1.027

npos 1.087 0.920 1.082 1.093

nex 0.966 1.035 0.962 0.971

Rsquare= 0.1 (max possible= 0.978 )

Likelihood ratio test= 2698 on 17 df, p=0

Wald test = 3029 on 17 df, p=0

Score (logrank) test = 3229 on 17 df, p=0

*** Generalized Additive Model ***

Call: gam(formula = count ~ s(AgeDgc) + s(EodSize) + s(npos) + s(nex) + s(YearDgc) + offset(log(newtime)),

family = poisson, data = m, na.action = na.exclude)

Deviance Residuals:

Min 1Q Median 3Q Max

-2.32205 -0.670597 -0.478176 -0.2440518 4.094334

(Dispersion Parameter for Poisson family taken to be 1 )

Null Deviance: 25044.75 on 25664 degrees of freedom

Residual Deviance: 22930.02 on 25643.98 degrees of freedom

Number of Local Scoring Iterations: 5

DF for Terms and Chi-squares for Nonparametric Effects

Df Npar Df Npar Chisq P(Chi)

(Intercept) 1

s(AgeDgc) 1 3 63.7252 0.00000000

s(EodSize) 1 3 78.6752 0.00000000

s(npos) 1 3 183.4774 0.00000000

s(nex) 1 3 34.9296 0.00000013

s(YearDgc) 1 3 7.7410 0.05190393

> cox.zph(fit2,transform='identity')

rho chisq p

seercent -0.00192 0.0189 8.91e-001

seerwest -0.01540 1.2192 2.70e-001

raceblck -0.00836 0.3565 5.50e-001

married -0.01269 0.8131 3.67e-001

innerkwd 0.01347 0.9154 3.39e-001

histduct -0.02923 4.3583 3.68e-002

erneg -0.07927 33.7642 6.22e-009

prneg -0.01169 0.7376 3.90e-001

g34 -0.07218 26.7764 2.28e-007

bcs -0.03030 4.6440 3.12e-002

rt 0.01995 2.0884 1.48e-001

bcrt 0.02141 2.3256 1.27e-001

YearDgc 0.02005 1.9934 1.58e-001

AgeDgc -0.00740 0.2984 5.85e-001

EodSize -0.06494 20.0007 7.74e-006

npos -0.03431 6.0313 1.41e-002

nex 0.03470 7.4047 6.51e-003

GLOBAL NA 186.7970 0.00e+000

### Breast-cancer specific cause of death

### N+, m$agetr <- m$AgeDgc+(abs(m$AgeDgc-50))^1.5

*** Cox Proportional Hazards ***

Call:

coxph(formula = Surv(futime, event2) ~ seercent + seerwest + raceblck + married + innerkwd +

histduct + erneg + prneg + g34 + bcs + rt + bcrt + YearDgc + agetr + EodSize + npos + nex,

data = m, na.action = na.exclude, method = "efron")

n= 25665

coef exp(coef) se(coef) z p

seercent -0.08459 0.919 0.037193 -2.274 2.3e-002

seerwest -0.15483 0.857 0.033751 -4.588 4.5e-006

raceblck 0.36893 1.446 0.045233 8.156 3.3e-016

married -0.07105 0.931 0.030220 -2.351 1.9e-002

innerkwd 0.26236 1.300 0.042579 6.162 7.2e-010

histduct 0.11645 1.124 0.035967 3.238 1.2e-003

erneg 0.46441 1.591 0.046413 10.006 0.0e+000

prneg 0.32775 1.388 0.043883 7.469 8.1e-014

g34 0.39085 1.478 0.029928 13.060 0.0e+000

bcs -0.00857 0.991 0.067393 -0.127 9.0e-001

rt -0.08185 0.921 0.042472 -1.927 5.4e-002

bcrt -0.13552 0.873 0.084870 -1.597 1.1e-001

YearDgc -0.06452 0.938 0.006446 -10.009 0.0e+000

agetr 0.00116 1.001 0.000233 4.963 6.9e-007

EodSize 0.02406 1.024 0.001278 18.836 0.0e+000

npos 0.08355 1.087 0.002525 33.095 0.0e+000

nex -0.03379 0.967 0.002420 -13.961 0.0e+000

exp(coef) exp(-coef) lower .95 upper .95

seercent 0.919 1.088 0.854 0.988

seerwest 0.857 1.167 0.802 0.915

raceblck 1.446 0.691 1.323 1.580

married 0.931 1.074 0.878 0.988

innerkwd 1.300 0.769 1.196 1.413

histduct 1.124 0.890 1.047 1.206

erneg 1.591 0.629 1.453 1.743

prneg 1.388 0.721 1.273 1.512

g34 1.478 0.676 1.394 1.568

bcs 0.991 1.009 0.869 1.131

rt 0.921 1.085 0.848 1.001

bcrt 0.873 1.145 0.739 1.031

YearDgc 0.938 1.067 0.926 0.949

agetr 1.001 0.999 1.001 1.002

EodSize 1.024 0.976 1.022 1.027

npos 1.087 0.920 1.082 1.093

nex 0.967 1.034 0.962 0.971

Rsquare= 0.1 (max possible= 0.978 )

Likelihood ratio test= 2715 on 17 df, p=0

Wald test = 3052 on 17 df, p=0

Score (logrank) test = 3247 on 17 df, p=0

*** Generalized Additive Model ***

Call: gam(formula = count ~ s(agetr) + s(EodSize) + s(npos) + s(nex) + s(YearDgc) + offset(log(newtime)),

family = poisson, data = m, na.action = na.exclude)

Deviance Residuals:

Min 1Q Median 3Q Max

-2.029272 -0.6709937 -0.4788156 -0.2439687 4.094292

(Dispersion Parameter for Poisson family taken to be 1 )

Null Deviance: 25060.7 on 25664 degrees of freedom

Residual Deviance: 22983.78 on 25643.99 degrees of freedom

Number of Local Scoring Iterations: 5

DF for Terms and Chi-squares for Nonparametric Effects

Df Npar Df Npar Chisq P(Chi)

(Intercept) 1

s(agetr) 1 3 9.2006 0.02675866

s(EodSize) 1 3 79.1750 0.00000000

s(npos) 1 3 182.2845 0.00000000

s(nex) 1 3 34.8635 0.00000013

s(YearDgc) 1 3 8.1735 0.04274886

> cox.zph(fit2,transform='identity')

rho chisq p

seercent -0.00147 0.0111 9.16e-001

seerwest -0.01567 1.2603 2.62e-001

raceblck -0.01006 0.5174 4.72e-001

married -0.01770 1.5954 2.07e-001

innerkwd 0.01268 0.8111 3.68e-001

histduct -0.02917 4.3422 3.72e-002

erneg -0.08062 34.8952 3.48e-009

prneg -0.01140 0.7009 4.02e-001

g34 -0.07325 27.4860 1.58e-007

bcs -0.03144 4.9946 2.54e-002

rt 0.01830 1.7615 1.84e-001

bcrt 0.02209 2.4757 1.16e-001

YearDgc 0.01987 1.9607 1.61e-001

agetr -0.02349 2.8087 9.38e-002

EodSize -0.06467 19.7169 8.98e-006

npos -0.03282 5.5247 1.87e-002

nex 0.03286 6.6477 9.93e-003

GLOBAL NA 189.1403 0.00e+000

### Breast-cancer specific cause of death

### N+, m$agetr <- m$AgeDgc+(abs(m$AgeDgc-50))^1.8

*** Cox Proportional Hazards ***

Call:

coxph(formula = Surv(futime, event2) ~ seercent + seerwest + raceblck + married + innerkwd +

histduct + erneg + prneg + g34 + bcs + rt + bcrt + YearDgc + agetr + EodSize + npos + nex,

data = m, na.action = na.exclude, method = "efron")

n= 25665

coef exp(coef) se(coef) z p

seercent -0.084498 0.919 0.037191 -2.272 2.3e-002

seerwest -0.154480 0.857 0.033752 -4.577 4.7e-006

raceblck 0.368634 1.446 0.045222 8.152 3.3e-016

married -0.069645 0.933 0.030245 -2.303 2.1e-002

innerkwd 0.262832 1.301 0.042580 6.173 6.7e-010

histduct 0.116288 1.123 0.035965 3.233 1.2e-003

erneg 0.463135 1.589 0.046378 9.986 0.0e+000

prneg 0.327960 1.388 0.043876 7.475 7.7e-014

g34 0.389962 1.477 0.029922 13.033 0.0e+000

bcs -0.010569 0.989 0.067357 -0.157 8.8e-001

rt -0.081273 0.922 0.042475 -1.913 5.6e-002

bcrt -0.133999 0.875 0.084854 -1.579 1.1e-001

YearDgc -0.064567 0.937 0.006446 -10.017 0.0e+000

agetr 0.000467 1.000 0.000091 5.129 2.9e-007

EodSize 0.023996 1.024 0.001277 18.787 0.0e+000

npos 0.083582 1.087 0.002524 33.118 0.0e+000

nex -0.033799 0.967 0.002419 -13.971 0.0e+000

exp(coef) exp(-coef) lower .95 upper .95

seercent 0.919 1.088 0.854 0.988

seerwest 0.857 1.167 0.802 0.915

raceblck 1.446 0.692 1.323 1.580

married 0.933 1.072 0.879 0.990

innerkwd 1.301 0.769 1.196 1.414

histduct 1.123 0.890 1.047 1.205

erneg 1.589 0.629 1.451 1.740

prneg 1.388 0.720 1.274 1.513

g34 1.477 0.677 1.393 1.566

bcs 0.989 1.011 0.867 1.129

rt 0.922 1.085 0.848 1.002

bcrt 0.875 1.143 0.741 1.033

YearDgc 0.937 1.067 0.926 0.949

agetr 1.000 1.000 1.000 1.001

EodSize 1.024 0.976 1.022 1.027

npos 1.087 0.920 1.082 1.093

nex 0.967 1.034 0.962 0.971

Rsquare= 0.1 (max possible= 0.978 )

Likelihood ratio test= 2716 on 17 df, p=0

Wald test = 3056 on 17 df, p=0

Score (logrank) test = 3250 on 17 df, p=0

*** Generalized Additive Model ***

Call: gam(formula = count ~ s(agetr) + s(EodSize) + s(npos) + s(nex) + s(YearDgc) + offset(log(newtime)),

family = poisson, data = m, na.action = na.exclude)

Deviance Residuals:

Min 1Q Median 3Q Max

-2.005416 -0.6714923 -0.478411 -0.2444962 4.088161

(Dispersion Parameter for Poisson family taken to be 1 )

Null Deviance: 25062.48 on 25664 degrees of freedom

Residual Deviance: 22986.31 on 25643.98 degrees of freedom

Number of Local Scoring Iterations: 5

DF for Terms and Chi-squares for Nonparametric Effects

Df Npar Df Npar Chisq P(Chi)

(Intercept) 1

s(agetr) 1 3 4.9151 0.1789048

s(EodSize) 1 3 79.2019 0.0000000

s(npos) 1 3 181.6262 0.0000000

s(nex) 1 3 34.7601 0.0000001

s(YearDgc) 1 3 8.0855 0.0444735

> cox.zph(fit2,transform='identity')

rho chisq p

seercent -0.00139 0.00983 9.21e-001

seerwest -0.01572 1.26854 2.60e-001

raceblck -0.01025 0.53830 4.63e-001

married -0.01878 1.80298 1.79e-001

innerkwd 0.01255 0.79455 3.73e-001

histduct -0.02915 4.33400 3.74e-002

erneg -0.08080 35.03623 3.24e-009

prneg -0.01140 0.70070 4.03e-001

g34 -0.07326 27.49975 1.57e-007

bcs -0.03157 5.03886 2.48e-002

rt 0.01795 1.69474 1.93e-001

bcrt 0.02216 2.49168 1.14e-001

YearDgc 0.01995 1.97557 1.60e-001

agetr -0.02683 3.64716 5.62e-002

EodSize -0.06445 19.56981 9.70e-006

npos -0.03266 5.46970 1.93e-002

nex 0.03260 6.54175 1.05e-002

GLOBAL NA 190.15153 0.00e+000
