# Supplementary material for: Modeling the effect of age in T1-2 breast cancer using the SEER database
Source: BMC Cancer. 2005 Oct 8;5:130. doi: 10.1186/1471-2407-5-130 (PMC1277821; doi:10.1186/1471-2407-5-130)
Supplement: Additional File 2 — GBSG-2 data set. [file 1471-2407-5-130-S2.doc]

Data set GBSG-2.

Node-positive

Event = recurrence or death

ht = hormone treatment

npos = number of positive nodes

sizetr = tumor size

pr = progesterone receptor

er = estrogen receptor

grad = histological grade

agetr = age covariate: untransformed,

transformed as age + |age-50|^1.5^, and transfromed as age+|age-50|1.8

summary of output

| **GBSG-2 data set** | no transform | transform  age+|age-50|1.5 | transform  age+|age-50|1.8 |
| --- | --- | --- | --- |
| Hazard ratio for Age | 1.001 | 1.004 | 1.002 |
| p-value of Hazard ratio for Age | 0.92 | 0.019 | 0.010 |
| GAM Chisq for Age | 31.74 | 9.29 | 8.70 |
| GAM p-value for Age | 0.0000006 | 0.0256 | 0.0336 |
| Test PH for Age: rho | 0.131 | 0.0459 | 0.0240 |
| Test PH for Age: chisq | 6.118 | 0.581 | 0.169 |
| Full PH model Rsquare | 0.135 | 0.142 | 0.143 |
| Full PH model Likelihood ratio test | 99.7 | 105 | 106 |

# No transform

*** Cox Proportional Hazards ***

Call:

coxph(formula = Surv(futime, event) ~ ht + agetr + sizetr + grad + npos + pr + er, data = m, na.action

= na.exclude, method = "efron")

n= 686

coef exp(coef) se(coef) z p

ht -0.321137 0.725 0.128655 -2.4961 1.3e-002

agetr 0.000625 1.001 0.006260 0.0998 9.2e-001

sizetr 0.007429 1.007 0.003922 1.8943 5.8e-002

grad 0.283062 1.327 0.105823 2.6749 7.5e-003

npos 0.050210 1.051 0.007403 6.7826 1.2e-011

pr -0.002306 0.998 0.000578 -3.9908 6.6e-005

er 0.000173 1.000 0.000444 0.3886 7.0e-001

exp(coef) exp(-coef) lower .95 upper .95

ht 0.725 1.379 0.564 0.933

agetr 1.001 0.999 0.988 1.013

sizetr 1.007 0.993 1.000 1.015

grad 1.327 0.753 1.079 1.633

npos 1.051 0.951 1.036 1.067

pr 0.998 1.002 0.997 0.999

er 1.000 1.000 0.999 1.001

Rsquare= 0.135 (max possible= 0.995 )

Likelihood ratio test= 99.7 on 7 df, p=0

Wald test = 113 on 7 df, p=0

Score (logrank) test = 118 on 7 df, p=0

*** Generalized Additive Model ***

Call: gam(formula = count ~ s(sizetr) + s(agetr) + s(npos) + s(er) + offset(log(newtime)), family = poisson,

data = m, subset = m$futime > 71, na.action = na.exclude)

Deviance Residuals:

Min 1Q Median 3Q Max

-2.164064 -0.9404463 -0.5607838 0.9232704 3.434006

(Dispersion Parameter for Poisson family taken to be 1 )

Null Deviance: 1001.316 on 671 degrees of freedom

Residual Deviance: 864.2169 on 655.1599 degrees of freedom

Number of Local Scoring Iterations: 6

DF for Terms and Chi-squares for Nonparametric Effects

Df Npar Df Npar Chisq P(Chi)

(Intercept) 1

s(sizetr) 1 3.0 7.37798 0.06070712

s(agetr) 1 3.0 31.74542 0.00000061

s(npos) 1 2.8 22.07435 0.00005054

s(er) 1 3.0 14.05065 0.00283826

> cox.zph(fit2,transform='identity')

rho chisq p

ht -0.0193 0.112 0.7375

agetr 0.1308 6.118 0.0134

sizetr -0.0263 0.208 0.6482

grad -0.1015 2.698 0.1005

npos 0.0680 0.783 0.3762

pr 0.0585 1.313 0.2518

er 0.0287 0.277 0.5985

GLOBAL NA 15.496 0.0301

# m$agetr <- m$age + (abs(m$age-50))^1.5

*** Cox Proportional Hazards ***

Call:

coxph(formula = Surv(futime, event) ~ ht + agetr + sizetr + grad + npos + pr + er, data = m, na.action

= na.exclude, method = "efron")

n= 686

coef exp(coef) se(coef) z p

ht -0.3551692 0.701 0.127513 -2.785 5.3e-003

agetr 0.0039697 1.004 0.001687 2.353 1.9e-002

sizetr 0.0080577 1.008 0.003923 2.054 4.0e-002

grad 0.2675780 1.307 0.105531 2.536 1.1e-002

npos 0.0485281 1.050 0.007404 6.554 5.6e-011

pr -0.0022557 0.998 0.000576 -3.918 8.9e-005

er -0.0000882 1.000 0.000449 -0.196 8.4e-001

exp(coef) exp(-coef) lower .95 upper .95

ht 0.701 1.426 0.546 0.900

agetr 1.004 0.996 1.001 1.007

sizetr 1.008 0.992 1.000 1.016

grad 1.307 0.765 1.063 1.607

npos 1.050 0.953 1.035 1.065

pr 0.998 1.002 0.997 0.999

er 1.000 1.000 0.999 1.001

Rsquare= 0.142 (max possible= 0.995 )

Likelihood ratio test= 105 on 7 df, p=0

Wald test = 120 on 7 df, p=0

Score (logrank) test = 125 on 7 df, p=0

*** Generalized Additive Model ***

Call: gam(formula = count ~ s(sizetr) + s(agetr) + s(npos) + s(er) + offset(log(newtime)), family = poisson,

data = m, subset = m$futime > 71, na.action = na.exclude)

Deviance Residuals:

Min 1Q Median 3Q Max

-2.039956 -0.9355048 -0.5681397 0.9666989 3.400911

(Dispersion Parameter for Poisson family taken to be 1 )

Null Deviance: 1003.182 on 671 degrees of freedom

Residual Deviance: 878.226 on 655.1862 degrees of freedom

Number of Local Scoring Iterations: 6

DF for Terms and Chi-squares for Nonparametric Effects

Df Npar Df Npar Chisq P(Chi)

(Intercept) 1

s(sizetr) 1 3.0 6.97989 0.07240305

s(agetr) 1 3.0 9.29372 0.02560622

s(npos) 1 2.8 21.49427 0.00006633

s(er) 1 3.0 15.30484 0.00157397

> cox.zph(fit2,transform='identity')

rho chisq p

ht -0.000126 4.91e-006 0.9982

agetr 0.045931 5.81e-001 0.4458

sizetr -0.017476 9.10e-002 0.7630

grad -0.116010 3.49e+000 0.0619

npos 0.073860 9.10e-001 0.3402

pr 0.046798 8.37e-001 0.3603

er 0.054497 1.04e+000 0.3089

GLOBAL NA 9.86e+000 0.1966

# m$agetr <- m$age + (abs(m$age-50))^1.8

*** Cox Proportional Hazards ***

Call:

coxph(formula = Surv(futime, event) ~ ht + agetr + sizetr + grad + npos + pr + er, data = m, na.action

= na.exclude, method = "efron")

n= 686

coef exp(coef) se(coef) z p

ht -0.3495484 0.705 0.127193 -2.748 6.0e-003

agetr 0.0018579 1.002 0.000722 2.573 1.0e-002

sizetr 0.0082219 1.008 0.003916 2.099 3.6e-002

grad 0.2625604 1.300 0.105495 2.489 1.3e-002

npos 0.0482711 1.049 0.007419 6.506 7.7e-011

pr -0.0022648 0.998 0.000576 -3.932 8.4e-005

er -0.0000833 1.000 0.000446 -0.187 8.5e-001

exp(coef) exp(-coef) lower .95 upper .95

ht 0.705 1.418 0.549 0.905

agetr 1.002 0.998 1.000 1.003

sizetr 1.008 0.992 1.001 1.016

grad 1.300 0.769 1.057 1.599

npos 1.049 0.953 1.034 1.065

pr 0.998 1.002 0.997 0.999

er 1.000 1.000 0.999 1.001

Rsquare= 0.143 (max possible= 0.995 )

Likelihood ratio test= 106 on 7 df, p=0

Wald test = 121 on 7 df, p=0

Score (logrank) test = 126 on 7 df, p=0

*** Generalized Additive Model ***

Call: gam(formula = count ~ s(sizetr) + s(agetr) + s(npos) + s(er) + offset(log(newtime)), family = poisson,

data = m, subset = m$futime > 71, na.action = na.exclude)

Deviance Residuals:

Min 1Q Median 3Q Max

-2.06772 -0.9404597 -0.5806465 0.9534044 3.421034

(Dispersion Parameter for Poisson family taken to be 1 )

Null Deviance: 1003.707 on 671 degrees of freedom

Residual Deviance: 877.9794 on 655.183 degrees of freedom

Number of Local Scoring Iterations: 6

DF for Terms and Chi-squares for Nonparametric Effects

Df Npar Df Npar Chisq P(Chi)

(Intercept) 1

s(sizetr) 1 3.0 7.04215 0.07040445

s(agetr) 1 3.0 8.69795 0.03360422

s(npos) 1 2.8 21.70359 0.00006015

s(er) 1 3.0 15.74656 0.00127740

> cox.zph(fit2,transform='identity')

rho chisq p

ht 0.00128 0.000505 0.9821

agetr 0.02401 0.168804 0.6812

sizetr -0.01885 0.105370 0.7455

grad -0.11504 3.424602 0.0642

npos 0.07702 0.988926 0.3200

pr 0.04534 0.786643 0.3751

er 0.06095 1.300293 0.2542

GLOBAL NA 9.438639 0.2227
